# Supplementary material for: Highly loaded bimetallic iron-cobalt catalysts for hydrogen release from ammonia
Source: Nat Commun. 2024 Jan 29;15:871. doi: 10.1038/s41467-023-44661-6 (PMC10824716; doi:10.1038/s41467-023-44661-6)
Supplement: Supplementary file 1 — Supplementary Information [file 41467_2023_44661_MOESM1_ESM.docx]

**Supplementary Information**

**Highly loaded Bimetallic Iron-Cobalt Catalysts for Hydrogen Release from Ammonia**

Shilong Chen^1^, Jelena Jelic^2^, Denise Rein^3,4^, Sharif Najafishirtari^1^, Franz-Philipp Schmidt^5^, Frank Girgsdies^5^, Liqun Kang^3^, Aleksandra Wandzilak^3^, Anna Rabe^1,4^_,_ Dmitry E. Doronkin^2,6^, Jihao Wang^1^, Klaus Friedel Ortega^1^, Serena DeBeer^3^, Jan-Dierk Grunwaldt^2,6^, Robert Schlögl^3,5^, Thomas Lunkenbein^5^, Felix Studt^2,6^, and Malte Behrens^1,4,7^*

^1^ Institute of Inorganic Chemistry, Kiel University,
Max-Eyth-Str. 2, 24118 Kiel, Germany

^2^ Institute of Catalysis Research and Technology, Karlsruhe Institute of Technology (KIT),
Hermann-von-Helmholtz-Platz 1, 76344 Eggenstein-Leopoldshafen, Germany

^3^ Max Planck Institute for Chemical Energy Conversion,
Stiftstrasse 34-36, 45470 Mülheim an der Ruhr, Germany

^4^ Faculty of Chemistry, University of Duisburg-Essen, Universtätsstr. 7, 45141 Essen, Germany

^5^ Fritz-Haber-Institut der Max-Planck-Gesellschaft, Department of Inorganic Chemistry,
Faradayweg 4-6, 14195 Berlin, Germany

^6^ Institute for Chemical Technology and Polymer Chemistry, Karlsruhe Institute of Technology (KIT), Engesserstr. 20, 76131 Karlsruhe, Germany

^7^ Kiel Nano, Surface and Interface Science KiNSIS, Kiel University, Christian-Albrechts-Platz 4, 24118 Kiel, Germany

* Corresponding author, email: [mbehrens@ac.uni-kiel.de](mailto:mbehrens@ac.uni-kiel.de)

**Table of Contents**

1. Weisz-Prater criterion / Mears’ criterion calculations pS3
2. Synthesis of Spinel pre-catalysts and basic characterizations pS4

Supplementary Fig. 1: Synthesis protocols

Supplementary Fig. 2: XRD patterns of precursors and spinel pre-catalysts

Supplementary Fig. 3: Thermogravimetric analysis

Supplementary Fig. 4: SEM and TEM images

Supplementary Fig. 5: Element analysis

Supplementary Table 1: BET specific surface area, pore volume and pore size

1. Reduction properties: H_2_-TPR in situ XRD pS8

Supplementary Fig. 6: H_2_-TPR

Supplementary Fig. 7: XRD patterns of metal catalysts

Supplementary Fig. 8-9: Rietveld refinements for the in situ XRD

Supplementary Table 2: The summary results from Rietveld refinement

1. XES of the Spent catalysts: Supplementary Fig. 10 pS11
2. Additional STEM-EDX map data: Supplementary Fig. 11 pS12
3. The procedure of EXAFS fitting and additional data pS13

Supplementary Fig. 12: XAFS data at Co K-edge

Supplementary Fig. 13: k^2^-weighted EXAFS functions in k space

Supplementary Fig. 14: Reference Fe K-edge EXAFS in k and R space

Supplementary Fig. 15: Activity data during reaction in XAFS reactor

Supplementary Fig. 16: Fitting results of k-space and R-space EXAFS spectra

Supplementary Table 3-4: Structural parameters

1. Additional catalytic data pS18

Supplementary Table 5: Comparison of the activity

Supplementary Fig. 17: NH_3_ conversion

Supplementary Fig. 18: Kinetics measurements

Supplementary Fig. 19: Stability test

Supplementary Fig. 20: Apparent activation energy

Supplementary Fig. 21: NH_3_ conversion under 10%NH_3_

Supplementary Fig. 22: Catalytic test and XRD of physically mixed Fe/MgO and Co/MgO

1. Metal particle size distribution and H_2_ Chemisorption pS22

Supplementary Fig. 23: TEM and metal particle size distribution

Supplementary Fig. 24: H_2_-TPD

1. Additional DFT information pS24

Supplementary Fig. 25: Phase diagram

Supplementary Table 6: Optimized lattice constants

Structures of the systems used in the Figure 4d

10. Supplementary References pS33

**1.** **Weisz-Prater criterion / Mears’ criterion calculations for mass transfer limitations for ammonia decomposition on Fe_1-x_Co_x_/MgO catalysts**

**1.1. Mass transfer limitations**: **Internal diffusion**, Weisz-Prater Criterion^[1](#_ENREF_1)^

The absence of internal mass transfer limitations can be verified by a Weisz-Prater criterion C_WP_ lower than 1.

$$C_{WP}=\frac{r_{abs}\rho_{c}R^{2}}{D_{eff}C_{As}}<1$$

r_abs_ = observed maximum reaction rate, mol kg_cat_^-1^ s^-1^ (here, the maximum NH_3_ reaction rate at 95.0% of NH_3_ conversion over Fe_1-x_Co_x_/MgO catalyst was used)

R = catalyst particle radius, m

*ρ_c_* = solid catalyst density, kg m^-3^

D*_eff_* = effective gas-phase diffusivity, m^2^ s^-1^

C_As_ = gas concentration of NH_3_ at the external surface of the catalyst, mol m^-3^

Hence, C_wp_ = {[0.0777 mol kg_cat_^-1^ s^-1^] × [4400 kg m^-3^] × [2×10^-4^ m]^2^}/ {[2.76×10^-5^ m^2^ s^-1^] × [1.23 mol m^-3^]} = 0.40 < 1

Therefore, the effect of internal mass transfer limitation on the catalytic measurements of ammonia decomposition can be neglected.

**1.2. Mass transfer limitations: External diffusion**, Mears’ criterion[^1^](#_ENREF_1)^,^[^2^](#_ENREF_2)

The absence of external mass transfer limitations can be verified by a Mears’ criterion C_M_ lower than 0.15:

$$C_{M}=\frac{r_{abs}\rho_{b}Rn}{k_{c}C_{Ab}}<0.15$$

r_abs_ = observed maximum reaction rate, mol kg_cat_^-1^ s^-1^ (here, the maximum NH_3_ reaction rate at 95.0% of NH_3_ conversion over Fe_1-x_Co_x_/MgO catalyst was used)

*ρ_b_* = bulk density of catalyst bed, kg m^-3^

R = catalyst particle radius, m

n = reaction order, using 0.6 as the maximum value for NH_3_ reaction order in ammonia decomposition

*k_c_* = external mass transfer coefficient, m s^-1^

*C_ab_* = bulk gas concentration of NH_3_, mol m^-3^

Hence, C_m_ = {[0.0777 mol kg_cat_^-1^ s^-1^] × [500 kg m^-3^] × [2×10^-4^ m] × 0.6}/ {[0.316 m s^-1^] × [1.23 mol m^-3^]} = 1.20 × 10^-2^ < 0.15

Therefore, the effect of external mass transfer limitation on the catalytic measurements of ammonia decomposition can be neglected.

**2. Synthesis of Spinel pre-catalysts and basic characterizations**

A series of Mg(Fe_1-x_Co_x_)_2_O_4_ spinel pre-catalysts with a variation of the Fe:Co ratio, including 1:0 (x = 0), 3:1 (x = 0.25), 1:1 (x = 0.5), and 0:1 (x = 1), were obtained from the calcination of layered double hydroxide precursors (LDHs) of the type Mg(Fe^II^_1-2x_Co_2x_)Fe^III^(OH)_6_(CO)_0.5_ ·*n* H_2_O for x = 0 – 0.5 and a Mg_1/3_Co_2/3_(OH)_2_ hydroxide precursor of the brucite structure type for x = 1. The precursors have been synthesized by a computer-controlled constant-pH co-precipitation (Supplementary Fig. 1). XRD (Supplementary Fig. 2a) showed that the recovered precursors consisted of both LDH and prematurely formed magnesioferrite spinel, MgFe_2_O_4_, for x = 0, 0.25, and 0.5 with a phase ratio depending on the Fe^2+^:Fe^3+^ ratio during synthesis.[^3^](#_ENREF_3) The precursor for x = 1 was crystallographically phase-pure (Mg,Co)(OH)_2_. According to thermogravimetry, a calcination temperature of 600°C in the air was sufficient to transform the precursor into a stable oxide (Supplementary Fig. 3). The morphology of the spinel pre-catalysts was inherited from the precursors with their layered structures resulting in a platelet-like morphology as seen in electron microscopy images (Supplementary Fig.4). Elemental analysis by AAS showed Fe:Co ratios of each sample that was in good agreement with the nominal values (Supplementary Fig. 5). The residual sodium content of all samples as measured by ICP-OES was around 0.20 ± 0.01 wt.% for the calcined samples. The specific surface areas determined by the BET method range from 14 to 87 m^2^ g^-1^ (Supplementary Table 1). The XRD patterns of x = 0 (MgFe_2_O_4_), x = 0.25 (Mg(Fe_0.75_Co_0.5_)_2_O_4_), and x = 0.5 (Mg(Fe_0.5_Co_0.5_)_2_O_4_) show reflections that can be assigned to the spinel structure of magnesioferrite (Supplementary Fig. 2b). This confirms that during calcination some of the Co^II^ and Fe^II^ species have been oxidized to their trivalent state giving rise to the A^II^B^III^_2_O_4_ spinel stoichiometry with A^II^ being Mg as the only irreducible species and B^III^ being reducible Fe-Co in different ratios. The (311) spinel reflection shifts to higher angles and becomes broader with increasing Co content supporting that it indeed is incorporated into the spinel lattice. The MgCo_2_O_4_ (x = 1) spinel pre-catalyst shows minor reflections of MgO as a by-phase. This small amount of segregated support phase is assumed to be negligible for the conclusions regarding the monometallic nature of this catalyst, i.e. the absence of Fe, in comparison with its bimetallic counterparts.


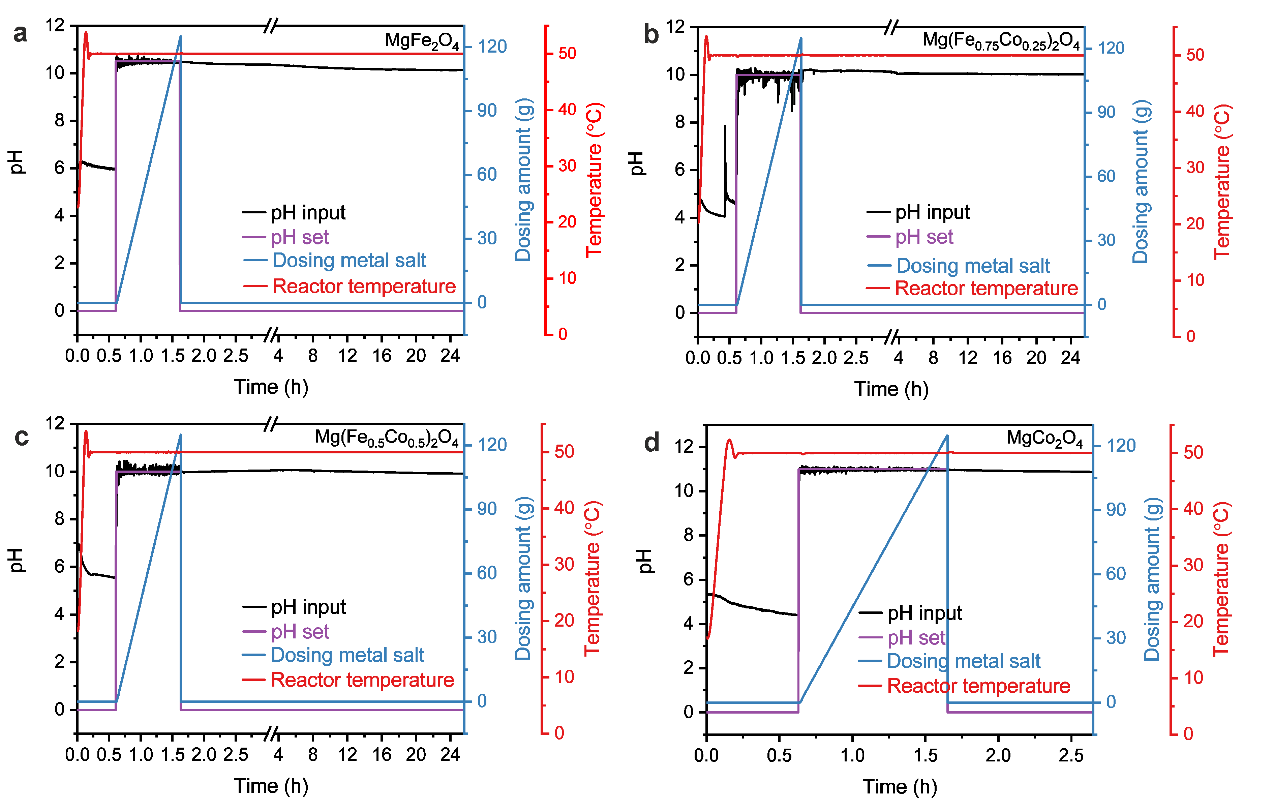


**Supplementary Fig. 1** **Synthesis recipe.** Synthesis protocols of pH and aging time variation series of MgFe_2_-pre (a), Mg(Fe_0.75_Co_0.25_)_2_-pre (b), Mg(Fe_0.5_Co_0.5_)_2_-pre (c) , and MgCo_2_-pre precursors (d).


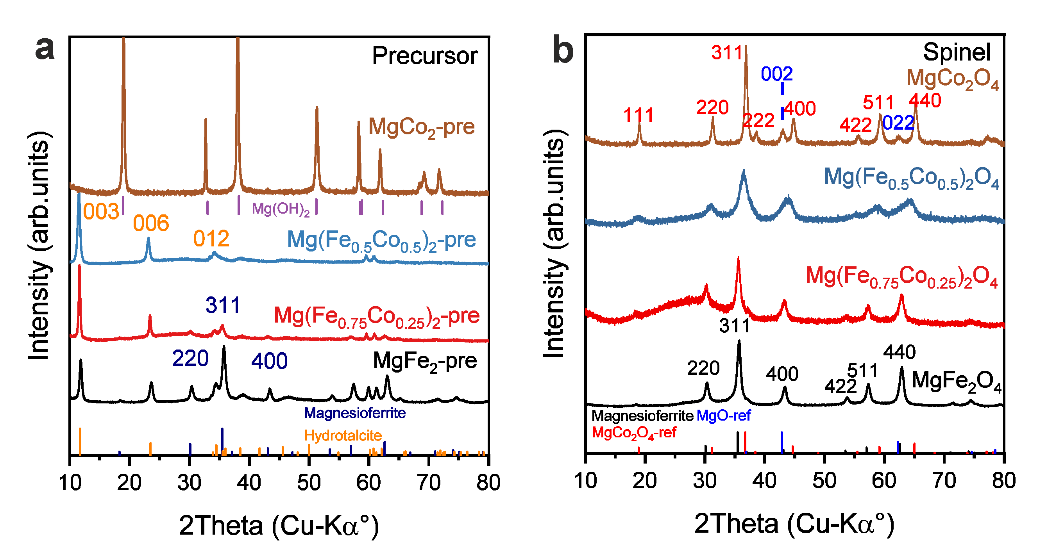


**Supplementary Fig. 2** **Structural characterization.** XRD patterns of Mg-Fe-Co based LDH/hydroxide precursors (a) and Mg-Fe-Co based spinel pre-catalysts (b). The references: Magnesioferrite (ICSD: 41290), Hydrotalcite (ICSD: 182294), MgO (ICSD: 9863), Mg(OH)_2_ (ICSD: 203213) and MgCo_2_O_4_ (ICSD: 190632).


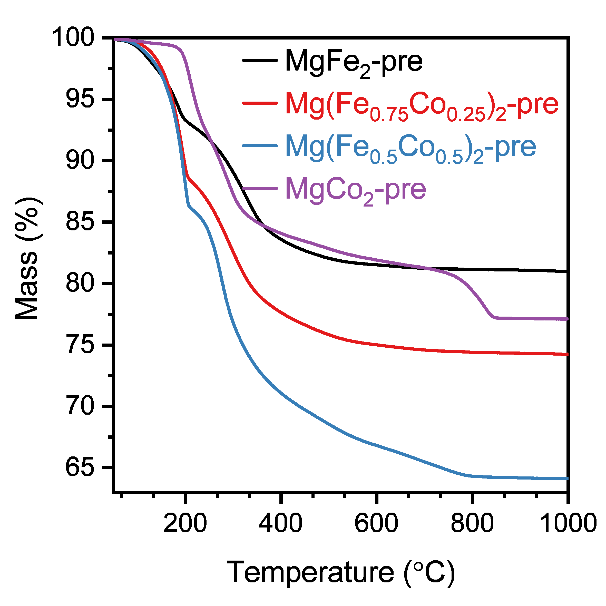


**Supplementary Fig. 3** **Composition analysis.** Thermogravimetric analysis of Mg-Fe-Co based LDH/hydroxide.


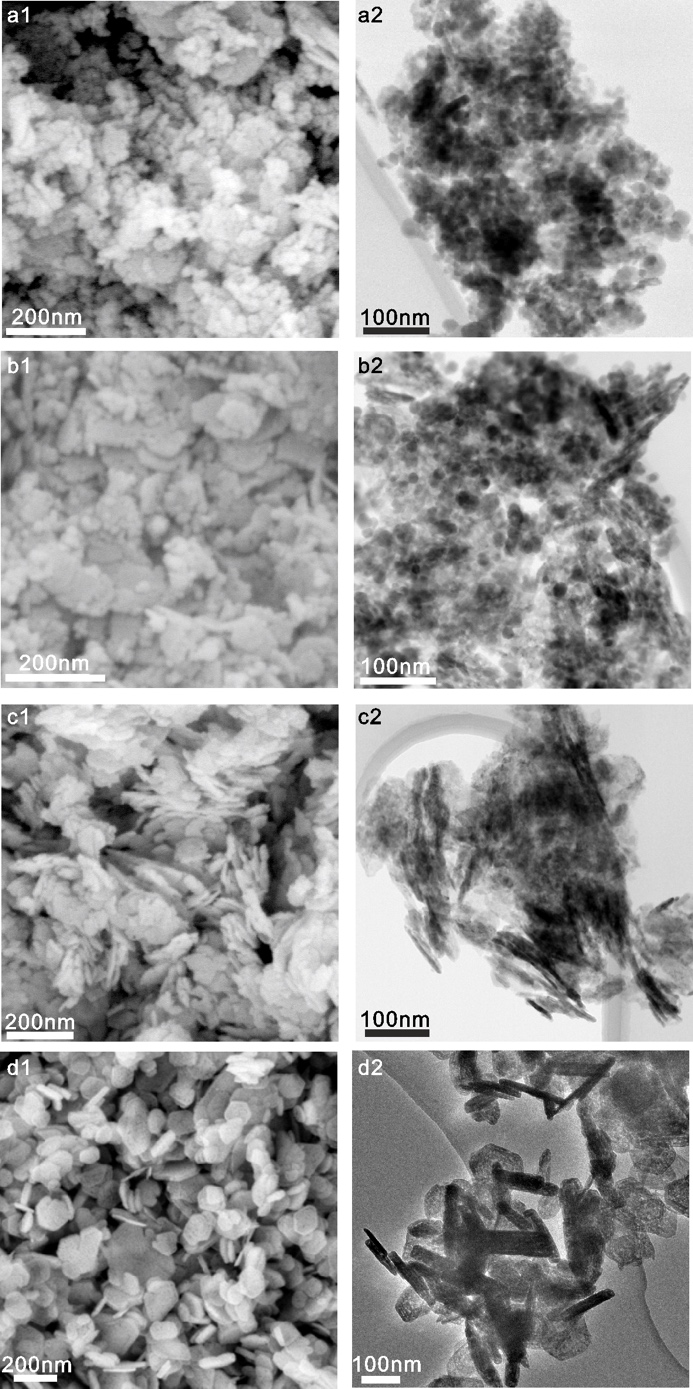


**Supplementary Fig. 4** **Morphology characterization.** Representative SEM (a1-d1) and TEM (a2-d2) images of MgFe_2_O_4_ (a1, a2), MgFe_1.5_Co_0.5_O_4_ (b1, b2), MgFeCoO_4_ (c1, c2), and MgCo_2_O_4_ (d1, d2) pre-catalysts.


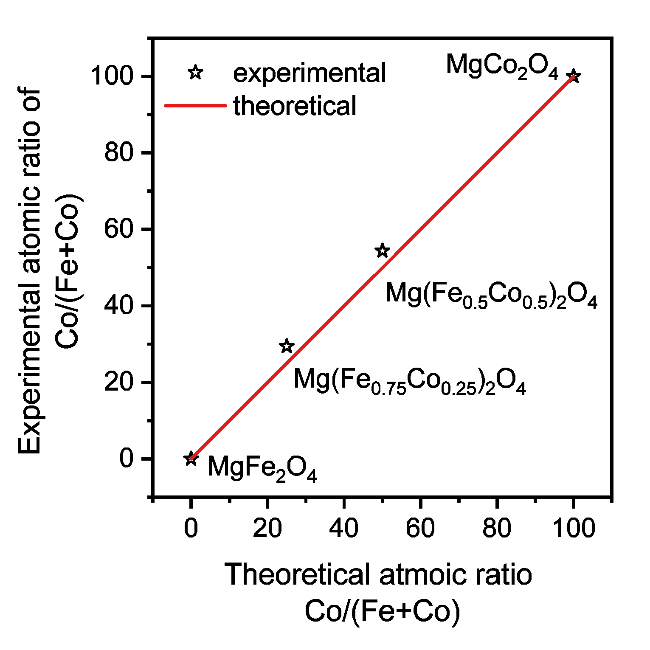


**Supplementary Fig. 5** **Elemental characterization.** Elemental analysis of the precursors of the four spinel pre-catalysts.

**Supplementary Table 1** BET specific surface area, pore volume and pore size of Mg-Fe-Co based LDH/hydroxide and the spinel pre-catalysts.

| *Catalyst* | *Surface area*  *(m^2^ g^-1^)* | *Pore volume*  *(cm^3^ g^-1^)* | *Average pore size*  *(nm)* |
| --- | --- | --- | --- |
| MgFe_2_-pre | 56.9 | 0.58 | 32.8 |
| MgFe_2_O_4_ | 75.7 | 0.55 | 8.8 |
| Mg(Fe_0.75_Co_0.25_)_2_-pre | 86.6 | 0.59 | 18.2 |
| Mg(Fe_0.75_Co_0.25_)_2_O_4_ | 85.4 | 0.51 | 25.8 |
| Mg(Fe_0.5_Co_0.5_)_2_-pre | 73.4 | 0.42 | 16.1 |
| Mg(Fe_0.5_Co_0.5_)_2_O_4_ | 70.0 | 0.77 | 26.0 |
| MgCo_2_-pre | 26.7 | 0.18 | 24.9 |
| MgCo_2_O_4_ | 13.8 | 0.06 | 3.40 |

**3. Reduction properties: H_2_-TPR and *in situ* XRD**


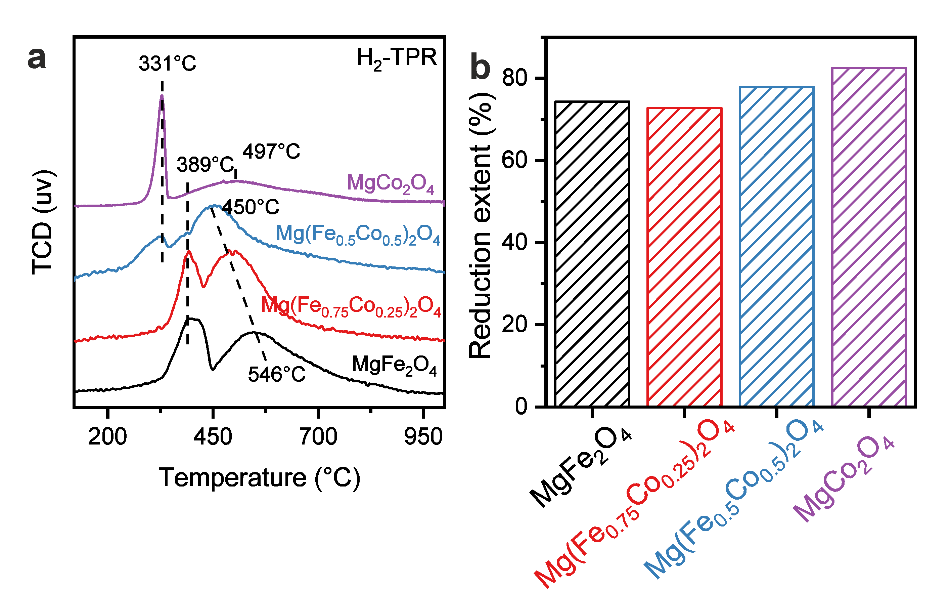


**Supplementary Fig. 6** **Reduction properties.** H_2_-TPR profiles of the four Mg(Fe_1-x_Co_x_)_2_O_4_ spinel pre-catalysts (a), and the reduction degree (%) of these spinel pre-catalysts (b).

MgFe_2_O_4_ shows typical two-reduction steps indicative of first Fe^III^ reduction in the spinel to Fe^II^ at 389°C forming a solid solution of (Mg_,_Fe)O, while the second TPR feature at 546°C was attributed to the formation of metallic Fe segregating from that mixed oxide. The degree of reduction of this spinel oxide after TPR at 1000 °C was estimated by the H_2_ consumption assuming that full reduction corresponds to a state with all iron/cobalt being metallic (Supplementary Fig. 6b) and was found to be around 70-80%. This indicates that a considerable fraction of iron is hard to reduce and remains present in the (Mg,Fe)O solid solution state. Based on the H_2_-TPR profile, an isothermal reduction temperature of 600°C for 5h was chosen for the activation of the spinel pre-catalyst.


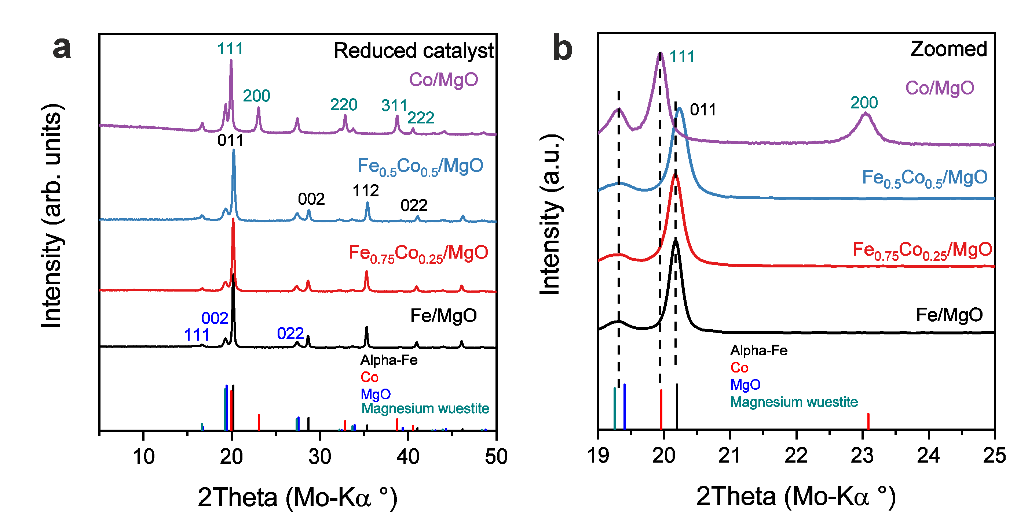


**Supplementary Fig. 7** **Structural characterization.** XRD patterns of reduced spinel catalysts after isothermal reduction in H_2_ at 600°C for 5h (a) and zoomed-in region from 2θ: 19-25° (b). The references: α-Fe (ICSD: 52258), MgO (ICSD: 9863), Magnesium wüstite (ICSD: 181215), fcc-Cobalt (PDF2: 00-015-0806). The resulting reduced catalysts were studied by XRD without exposure to air.


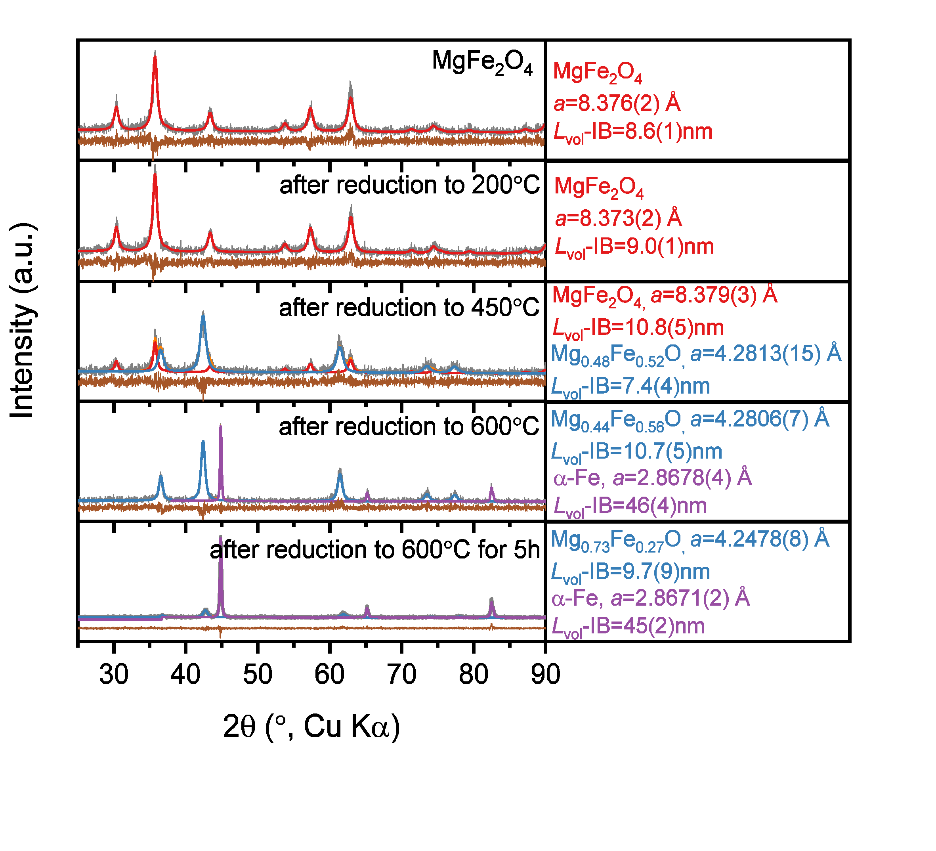


**Supplementary Fig. 8** **Composition analysis.** Rietveld refinements for the *in situ* XRD patterns of the activation process of MgFe_2_O_4_ under 7% H_2_/Ar. Measured data is shown in grey, the calculated patterns are in red (MgFe_2_O_4_), blue (MgFeO, iron-magnesium-wüstite), purple (α-Fe). The lattice parameters obtained from Rietveld refinement and the crystallite sizes (L_vol_-IB) determined as the volume-weight mean column height from integral breadth are summarized on the right.


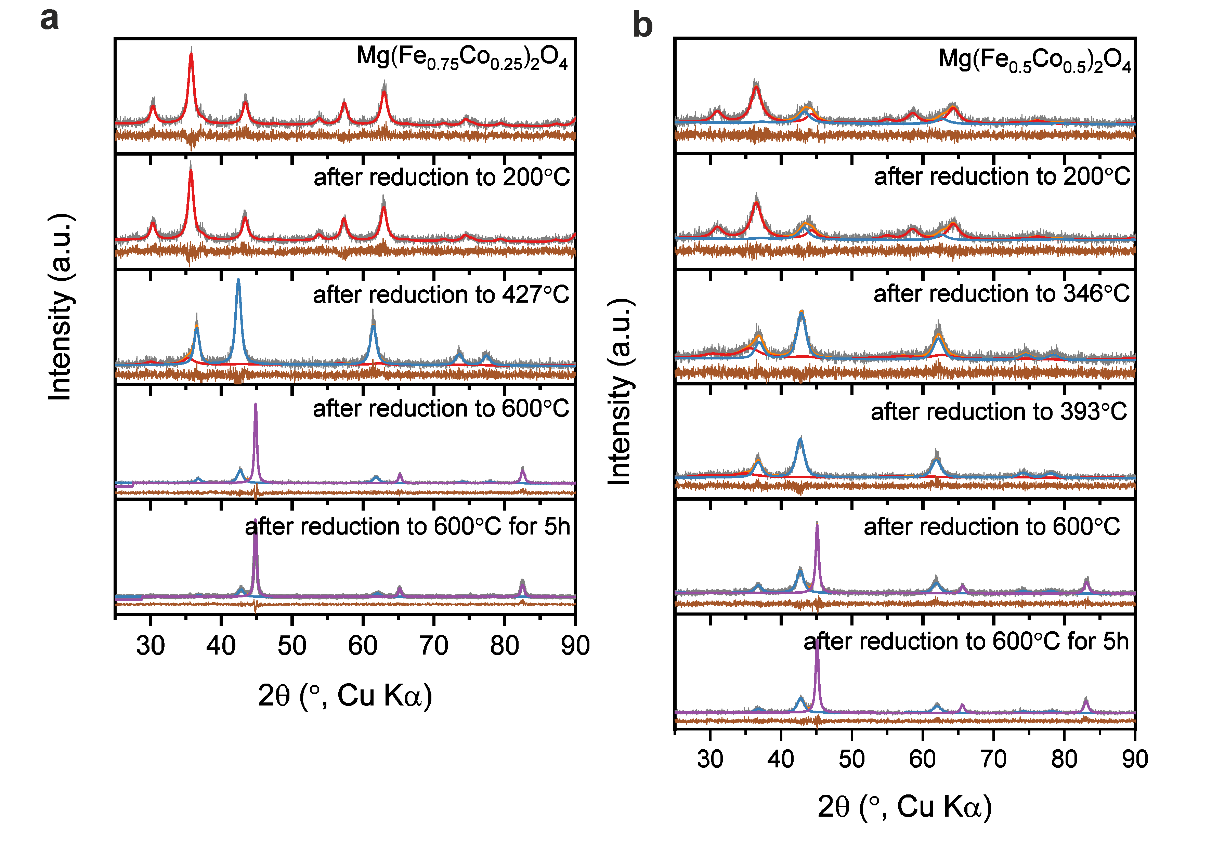


**Supplementary Fig. 9** **Composition analysis.** Rietveld refinements for the *in situ* XRD patterns of the activation process of Mg(Fe_0.75_Co_0.25_)_2_O_4_ (a) and Mg(Fe_0.5_Co_0.5_)_2_O_4_ (b) under 7% H_2_/Ar. Measured data is shown in grey, the calculated patterns are in red (Mg(Fe_,_Co)_2_O_4_), blue (Mg(Fe,Co)O, iron-magnesium-wüstite), purple (bcc-FeCo alloy).

**Supplementary Table 2** The summary results from Rietveld refinement, including component, lattice parameter, and crystallite size.

| ***Activation processes*** | ***Components*** | ***Percentage (%)*** | ***Lattice parameter (Å)*** | ***Crystallite size (nm)*** |
| --- | --- | --- | --- | --- |
| ***MgFe_2_O_4_*** | | | | |
| **MgFe_2_O_4_ as synthesized** | MgFe_2_O_4_ | 100 | 8.376(2) | 8.6(1) |
| **Reduction to 200°C** | MgFe_2_O_4_ | 100 | 8.373(2) | 9.0(1) |
| **Reduction to 450°C** | MgFe_2_O_4_ | 26.2 | 8.379(3) | 10.8(5) |
|  | Mg_0.48_Fe_0.52_O | 73.8 | 4.2813(15) | 7.4(4) |
| **Reduction to 600°C** | Mg_0.44_Fe_0.56_O | 87.2 | 4.2806(7) | 10.7(5) |
|  | α-Fe | 12.8 | 2.8678(4) | 46(4) |
| **Keeping at 600°C  for 5h** | Mg_0.73_Fe_0.27_O | 46.8 | 4.2478(8) | 9.7(9) |
|  | α-Fe | 53.2 | 2.8671(2) | 45(2) |
| ***Mg(Fe_0.75_Co_0.25_)_2_O_4_*** | | | | |
| **Mg(Fe_0.75_Co_0.25_)_2_O_4_ as synthesized** | Mg(Fe,Co)_2_O_4_ | 100 | 8.364(2) | 7.0(1) |
| **Reduction to 200°C** | Mg(Fe,Co)_2_O_4_ | 100 | 8.371(2) | 7.2(1) |
| **Reduction to 427°C** | Mg(Fe,Co)_2_O_4_ | 13.5 | 8.448(13) | 3.4(4) |
|  | Mg_0.40_(Fe,Co)_0.60_O | 86.5 | 4.2760(13) | 14.5(6) |
| **Reduction to 600°C** | Mg_0.59_(Fe,Co)_0.41_O | 48.2 | 4.2508(9) | 7.8(6) |
|  | (Fe,Co)-bcc | 51.8 | 2.8656(4) | 19.7(3) |
| **Keeping at 600°C  for 5h** | Mg_0.77_(Fe,Co)_0.23_O | 39.6 | 4.2374(10) | 7.6(8) |
|  | (Fe,Co)-bcc | 60.4 | 2.8663(3) | 21.6(3) |
| ***Mg(Fe_0.5_Co_0.5_)_2_O_4_*** | | | | |
| **Mg(Fe_0.5_Co_0.5_)_2_O_4_ as synthesized** | Mg(Fe,Co)_2_O_4_ | 69.0 | 8.220(6) | 3.6(8) |
|  | Mg(Fe,Co)O | 31.0 | 4.202(5) | 3.4(2) |
| **Reduction to 200°C** | Mg(Fe,Co)_2_O_4_ | 71.2 | 8.218(6) | 3.5(8) |
|  | Mg(Fe,Co)O | 28.8 | 4.206(5) | 3.7(3) |
| **Reduction to 346°C** | Mg(Fe,Co)_2_O_4_ | 36.0 | 8.380(16) | 1.7(11) |
|  | Mg_0.55_(Fe,Co)_0.45_O | 64.0 | 4.227(3) | 4.8(3) |
| **Reduction to 393°C** | Mg(Fe,Co)_2_O_4_ | 15.4 | 8.57(3) | 1.6(16) |
|  | Mg_0.47_(Fe,Co)_0.53_O | 84.6 | 4.243(2) | 5.2(2) |
| **Reduction to 600°C** | Mg_0.58_(Fe,Co)_0.42_O | 66.2 | 4.2445(12) | 10.5(7) |
|  | (Fe,Co)-bcc | 33.8 | 2.8486(6) | 14.2(3) |
| **Keeping at 600°C  for 5h** | Mg_0.69_(Fe,Co)_0.31_O | 55.6 | 4.2404(11) | 6.2(5) |
|  | (Fe,Co)-bcc | 44.4 | 2.8510(5) | 16.6(3) |

**4. XES of the spent catalysts**


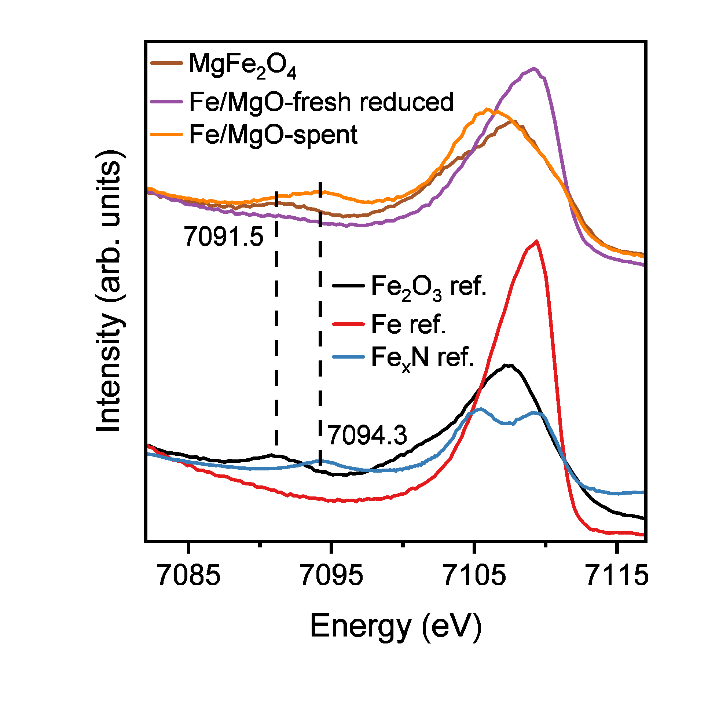


**Supplementary Fig. 10** **XES characterization.** Experimental Fe K*β* XES spectra in the valence-to-core (VtC) region of MgFe_2_O_4_ (dark yellow curve), Fe/MgO-fresh reduced (violet curve) and Fe/MgO-spent after ammonia decomposition (orange curve), as well as Fe_2_O_3_ (black curve), Fe metal (red curve) and Fe_x_N (blue curve) for references.

X-ray Emission Spectroscopy (XES) characterization, as a complementary technique to XAS, provides additional insights into the electronic structure of the MgFe_2_O_4_ catalysts. In particular, the valence-to-core (VtC) XES is primarily sensitive to the electronic structure of the ligands and has been used to probe the ligand identity[^4-6^](#_ENREF_4). Here in the VtC region of Fe K*β* XES (Supplementary Fig. 10), two characteristic features, K*β″* (7085-7100 eV) and K*β*_2,5_ (7100-7115 eV) are observed, corresponding to transitions from molecular orbitals of predominantly ligand *n*s and *n*p character to the Fe 1s core hole, respectively[^5^](#_ENREF_5)^,^[^7^](#_ENREF_7). As the ligand *n*s orbitals negligibly bond with the metal center, the K*β″* peak serves as ideal characteristic feature of the coordinating atoms with minimal modulations in the energy of the features due to changes in geometry and bond lengths, and is especially powerful enough to resolve the first row atoms due to the large variations of their ionization potentials[^8^](#_ENREF_8). In this case, both calcined MgFe_2_O_4_ catalyst and Fe_2_O_3_ reference show K*β″* features at around 7091.5 eV resulting from refilling of Fe 1s by electrons in the ligand O 2s orbitals. After H_2_ reduction, no obvious K*β″* peak could be observed for the catalyst, suggesting the removal of O from the Fe centers and formation of metallic Fe. Most interestingly, upon exposure to NH_3_ for the decomposition reaction, the spent catalyst exhibits a new and unique K*β″* signal at 7094.3 eV. Comparing the Fe_x_N reference material which has a same K*β″* feature at 7094.2 eV, it is concluded that a significant amount of Fe species in the spent catalysts are coordinated with N atoms, consistent with the formation of an iron nitride phase. Meanwhile, the energy shift of K*β″* peak of the spent catalyst to higher energies than that of the calcined catalyst is in good agreement with the fact that N 2s electrons have lower ionization potentials than the O 2s electrons.

**5. Additional STEM-EDS map data**


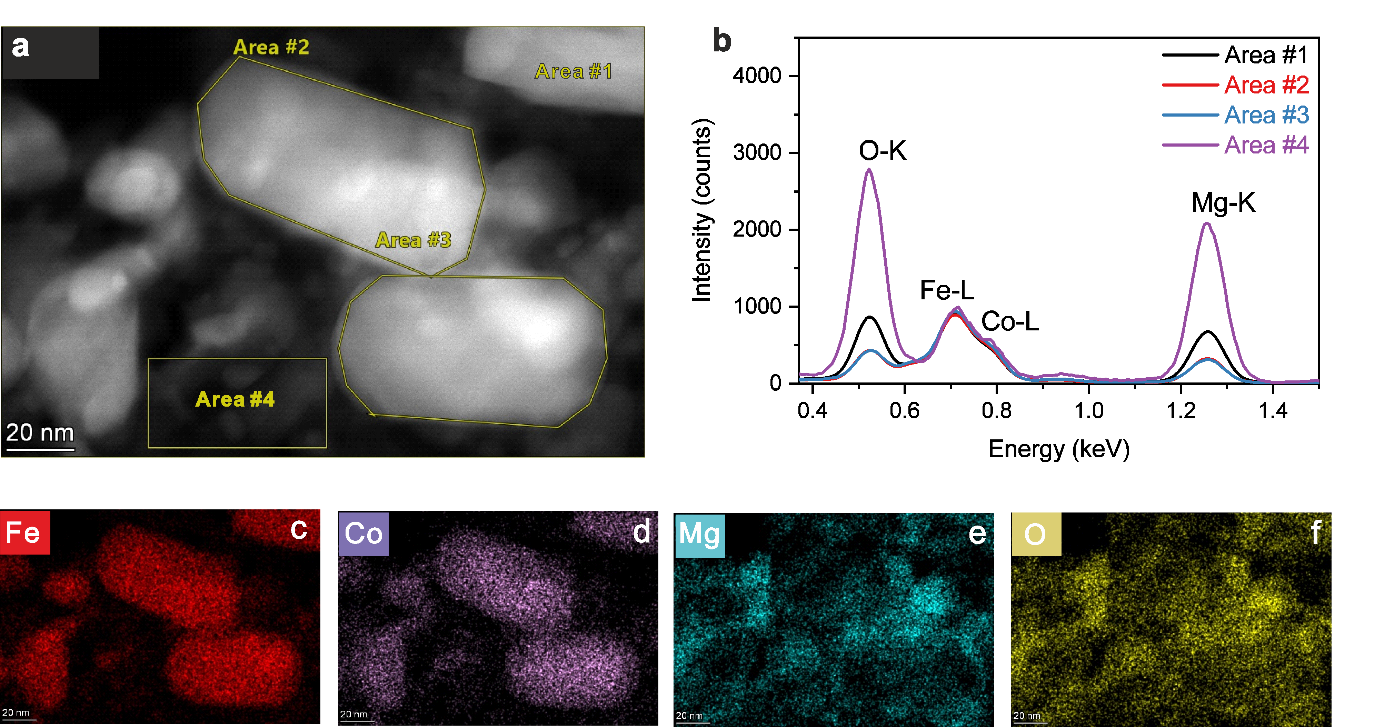


**Supplementary Fig. 11**  **STEM-EDS-map characterization.** STEM image of Fe_0.75_Co_0.25_/MgO catalyst (a) and the corresponding EDX spectra collected at Area #1, #2, #3, #4 (b), as well as EDX maps, which are related to K-line intensities from Fe (c), Co (d), Mg (e), and O (f). Note that the intensity (counts) of these four areas were normalized to the highest intensity of Fe-L peak.

**6. The procedure of EXAFS fitting and additional data**

XAFS data was analyzed using the Demeter software package (including Athena and Artemis software, version 0.9.26)[^9^](#_ENREF_9). Pre-edge background subtraction, post-edge normalization and forward Fourier Transform of the XAS data were processed by the Athena software. A linear regression background (6960-7070 eV) was determined, and a quadratic polynomial regression for post-edge normalization was applied (7260-7910 eV). The spectra were splined from *k*=0 to around 11-13 Å^-1^ (depending on the data quality) with rbkg set to 1.0 or 1.1. The *k*^2^-weighted k-space EXAFS spectra were Fourier Transformed to R-space using a Hanning window with d*k* = 1 (from *k*=3 to around 10-12 Å^-1^ depending on the data quality). The *k*^2^-weighted R-space EXAFS spectra were fitted using the Artemis software based on scattering paths generated from FEFF6 (built-in program in Artemis). Due to the limited data quality obtained from the *operando* XAS experiment, only the EXAFS spectra for Fe/MgO catalysts were quantitatively analyzed. The fitting results are listed in Supplementary Table 3.


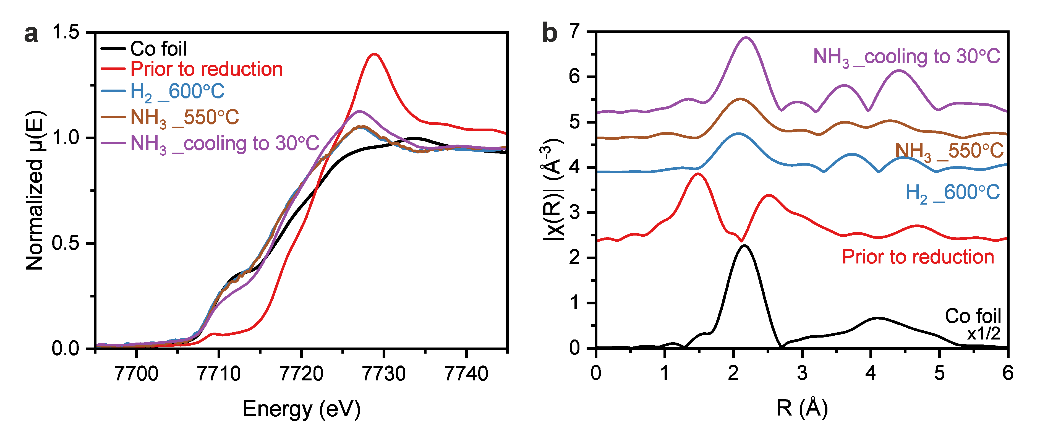


**Supplementary Fig. 12** **Operando XAS.** Co K-edge XAS spectra of Fe_0.5_Co_0.5_/MgO catalyst. (a) XANES spectra (normalized) and (b) *k*^2^-weighted R-space EXAFS spectra (plotted without phase correction). The Co foil EXAFS spectra in (b) is plotted with ½ intensity. The corresponding k-space EXAFS spectra are plotted in Supplementary Fig. 13(c). Note: the metallic FeCo phase formed at high temperatures has *bcc* structure instead of the *hcp* structure for monometallic Co foil. Therefore, their XANES and EXAFS features are not identical.


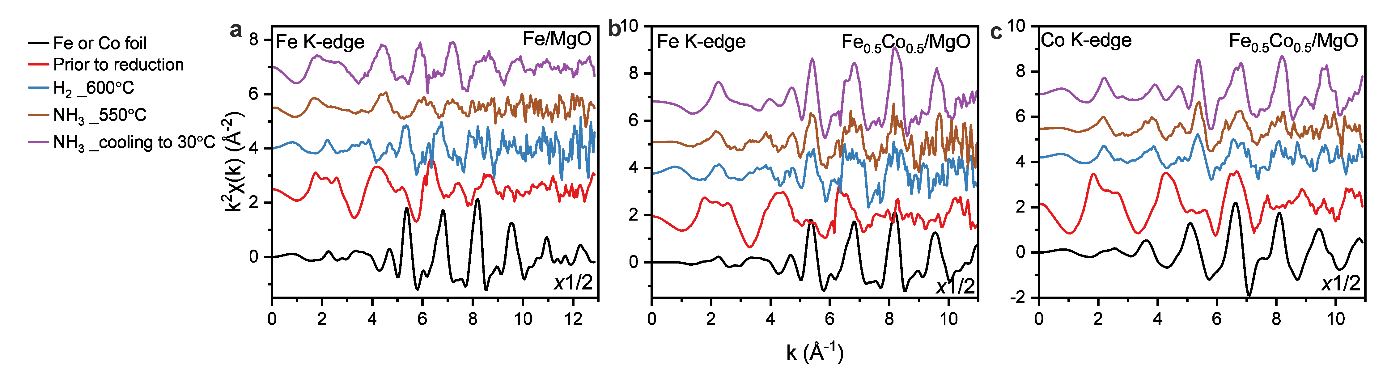


**Supplementary Fig. 13** **Operando XAS.** Fe K-edge and Co K-edge k^2^-weighted k-space EXAFS spectra. (a) Fe K-edge EXAFS spectra of Fe/MgO catalyst. (b) Fe K-edge EXAFS spectra of Fe_0.5_Co_0.5_/MgO catalyst. (c) Co K-edge EXAFS spectra of MgFeCo catalyst. The Fe foil and Co foil EXAFS spectra are plotted with ½ intensity.


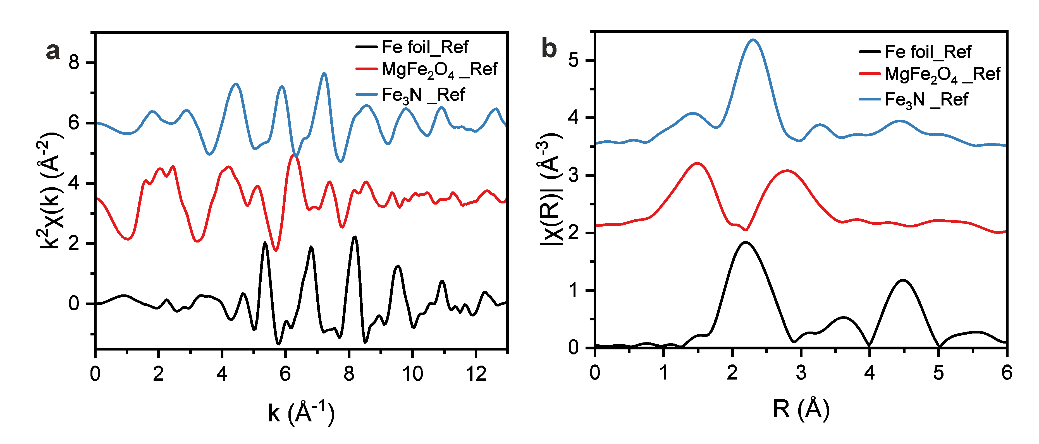


**Supplementary Fig. 14** **EXAFS references.** Reference Fe K-edge EXAFS spectra of Fe metal, MgFe_2_O_4_ and Fe_3_N. (a) *k*^2^-weighted *k*-space EXAFS spectra. (b) *k*^2^-weighted R-space EXAFS spectra (plotted without phase correction). The Fe foil EXAFS spectra are plotted with ½ intensity. These reference spectra were retrieved from the SPring-8 BL14B2 XAFS Standard Sample Database.


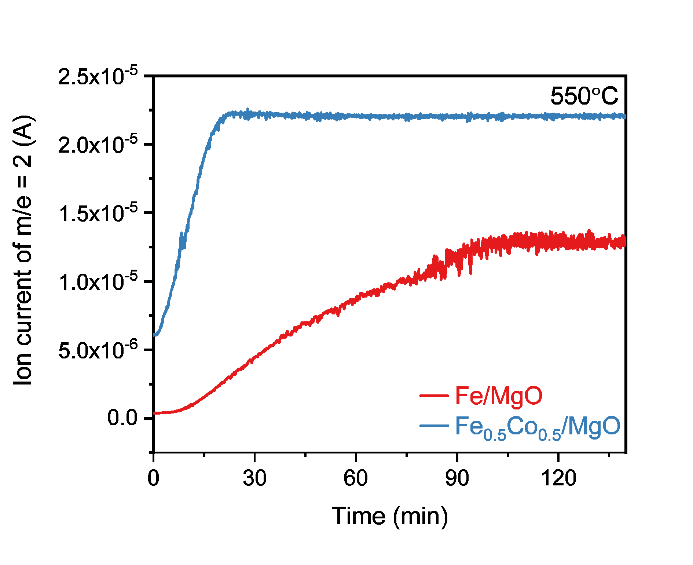


**Supplementary Fig. 15** **Catalytic Activity in operando XAS measurements.** Activity of ammonia decomposition on Fe/MgO and Fe_0.5_Co_0.5_/MgO catalysts at 550°C. The data were collected by mass spectrometer from the XAFS reactor (quartz capillary micro reactor[^10^](#_ENREF_10)) during the XAS measurements.

The Fe/MgO catalyst showed a longer activation phase than the Fe_0.5_Co_0.5_/MgO catalyst, which is due to the nitridation process on the iron catalyst.





**Supplementary Fig. 16** **EXFAS fitting.** Fitting results of k^2^-weighted k-space and R-space EXAFS spectra for (a,b) Fe foil, (c,d) MgFe_2_O_4_ pre-catalyst before reduction at room temperature, (e,f) Fe/MgO catalyst under H_2_ reduction at 600 °C, (g,h) Fe/MgO catalyst during NH_3_ decomposition at 550 °C; (i,j) Fe/MgO catalyst after NH_3_ decomposition at 550 °C and cooled down to 30°C. The fitting results are listed in Supplementary Table 3. The R-space EXAFS spectra are plotted without phase correction.

**Supplementary Table 3**. Fitting results of Fe K-edge *k*^2^-weighted R-space EXAFS spectra for Fe/MgO catalyst.*^a^*

| Sample | Scattering path | S_0_^2^ (amp) | CN | R [Å] | Rref [Å] | σ_2_ [Å^2^] | ΔE_0_ [eV] | R-factor |
| --- | --- | --- | --- | --- | --- | --- | --- | --- |
| Fe foil | Fe-Fe | 0.653 ± 0.059 | 8 (fixed) | 2.46 ± 0.01 | 2.48 | 0.0046 ± 0.0007 | 4.9 ± 1.1 | 0.0060 |
|  | Fe-Fe |  | 6 (fixed) | 2.84 ± 0.01 | 2.87 |  |  |  |
| Prior to reduction *^b^* | Fe-O | 0.653 (fixed) | 4.4 ± 0.4 | 1.92 ± 0.01 | 2.00 | 0.0053 ± 0.0012 | -1.9 ± 1.4 | 0.0090 |
|  | Fe-Fe |  | 4.2 ± 1.3 | 3.04 ± 0.02 | 2.96 | 0.0103 ± 0.0034 |  |  |
|  | Fe-Fe |  | 4.0 ± 1.4 | 3.50 ± 0.02 | 3.47 |  |  |  |
| H_2__600°C*^c^* | Fe-Fe | 0.653 (fixed) | 4.3 ± 0.8 | 2.47 ± 0.01 | 2.48 | 0.0123 ± 0.0022 | 4.1 ± 2.0 | 0.0165 |
|  | Fe-Fe |  | 2.1 ± 0.9 | 2.86 ± 0.01 | 2.87 |  |  |  |
| NH_3__550°C*^d^* | Fe-N/O | 0.653 (fixed) | 1.5 ± 0.2 | 2.04 ± 0.02 | 1.92 | 0.0076 ± 0.0012 | 0.6 ± 2.1 | 0.0084 |
|  | Fe-Fe |  | 3.7 ± 0.7 | 2.73 ± 0.02 | 2.71 | 0.0151 ± 0.0024 |  |  |
| NH_3__cooling to 30°C *^d^* | Fe-N/O | 0.653 (fixed) | 1.8 ± 0.3 | 1.95 ± 0.02 | 1.92 | 0.0034 ± 0.0007 | 0.1 ± 1.3 | 0.0075 |
|  | Fe-Fe |  | 4.6 ± 0.7 | 2.72 ± 0.01 | 2.71 | 0.0068 ± 0.0015 |  |  |

*^a^* S_0_^2^ = amplitude reduction factor, this value (0.653) is determined by fitting the EXAFS of Fe foil and used as a fixed parameter for the EXAFS fitting of other Fe samples; CN = coordination number; R = interatomic distance; R_ref_ = interatomic distances of reference materials. The interatomic distances in Fe metal, MgFe_2_O_4_ and Fe_3_N are obtained from the standard crystal structure retrieved from the Crystal Open Database (entry ID of Fe: 9008536; entry ID of MgFe_2_O_4_: 1011245, entry ID Fe_3_N: 2310869). σ^2^ = Debye-Waller factor. ΔE_0_ = energy shift refers to the E_0_ position in the EXAFS fitting model. The fitting errors of CN are mathematically calculated by the Artemis program. Due to the strong correlation between CN and σ^2^, the actual errors of CN would be affected by the uncertainties of the σ^2^ values and be bigger than the program-estimated values. Due to the data quality from the operando experiment, the total number of variables in the EXAFS fitting model was limited to a minimum for only necessary scattering paths.

*^b^* For the MgFe_2_O_4_ pre-catalyst before H_2_ reduction, one Fe-O scattering path was included for the Fe-O coordination of Fe species in both O_h_ and T_d_ sites. As Mg is a much lighter scatterer than Fe, the Fe-Mg scattering paths were not included in the fitting model.

*^c^* For the Fe/MgO catalyst after H_2_ reduction, two Fe-Fe scattering paths were considered to represent the α-Fe with *bcc* structure.

*^d^* For the Fe/MgO catalyst during/after NH_3_ decomposition, the two scattering paths Fe-N/O and Fe-Fe were included to represent the contribution from light and heavy scatterers, respectively. Due to the similarity between N and O as back scatterers, it is not possible to distinguish between Fe-N and Fe-O scattering paths with the same (or similar) interatomic distance(s).

**Supplementary Table 4.** FEFF calculated coordination number and bond length of Fe reference compounds.

| Compound | Space Group | Path | C.N. | R [Å] | Ref. | COD ID* |
| --- | --- | --- | --- | --- | --- | --- |
| α-Fe | $Im\bar{3}m$ | Fe-Fe | 8 | 2.482 | [^11^](#_ENREF_11) | 9008536 |
|  |  | Fe-Fe | 6 | 2.867 |  |  |
| MgFe_2_O_4_ | $Fd\bar{3}m:1$ | Fe(O_h_)-O | 6 | 2.090 | [^12^](#_ENREF_12) | 1011245 |
|  |  | Fe(O_h_)-Mg/Fe | 6 | 2.956 |  |  |
|  |  | Fe(O_h_)-Mg/Fe | 6 | 3.466 |  |  |
|  |  | Fe(T_d_)-O | 4 | 1.810 |  |  |
|  |  | Fe(T_d_)-Mg/Fe | 12 | 3.466 |  |  |
| FeO | $Fm\bar{3}m$ | Fe-O | 6 | 2.155 | [^13^](#_ENREF_13) | 9008636 |
|  |  | Fe-Fe | 12 | 3.048 |  |  |
| FeN | $F\bar{4}3m$ | Fe-N | 4 | 1.865 | [^14^](#_ENREF_14) | 1535284 |
|  |  | Fe-Fe | 12 | 3.046 |  |  |
| Fe_2_N | $P312$ | Fe-N | 3 | 1.941 | [^15^](#_ENREF_15) | 2310870 |
|  |  | Fe-Fe | 6 | 2.726 |  |  |
|  |  | Fe-Fe | 6 | 2.764 |  |  |
| Fe_3_N | $P312$ | Fe-N | 2 | 1.918 | [^15^](#_ENREF_15) | 2310869 |
|  |  | Fe-Fe | 12 | 2.712 |  |  |
| Fe_4_N | $P\bar{4}3m$ | Fe-N | 1 | 1.740 | [^16^](#_ENREF_16) | 9004225 |
|  |  | Fe-Fe | 3 | 2.519 |  |  |
|  |  | Fe-Fe | 6 | 2.685 |  |  |
|  |  | Fe-Fe | 3 | 2.841 |  |  |

* Reference lattice parameters are retrieved from the *Crystallography Open Database* (<http://www.crystallography.net/>).[^17^](#_ENREF_17) [^18^](#_ENREF_18)

**7. Additional catalytic data**

**Supplementary Table 5** Comparison of catalytic performances of Fe-based and Ru-based catalysts in the ammonia decomposition reaction.

| Catalyst | Reaction temperature / °C | Space velocity / mL_NH3_ g^-1^_cat_ h^-1^ | Reaction rate / mol_H2_ g^-1^_cat_ h^-1^ | Ref. |
| --- | --- | --- | --- | --- |
| Fe/MgO | 500 | 7200 | 0.21 | This work |
| Fe/CNT | 500 | 30000 | 0.04 | Ref[^19^](#_ENREF_19) |
| Fe/Alumina matrix | 500 | 9000 | 0.33 | Ref[^20^](#_ENREF_20) |
| Fe-carbon nanofiber, mica | 500 | 6500 | 0.17 | Ref[^21^](#_ENREF_21) |
| Fe/CMK-15 carbon | 500 | 7500 | 0.11 | Ref[^22^](#_ENREF_22) |
| Fe/YSZ | 550 | 46000 | 0.16 | Ref[^23^](#_ENREF_23) |
| Ru/MgO | 450 | 30000 | 1.13 | Ref[^24^](#_ENREF_24) |
| Ru/CNT | 500 | 30000 | 1.70 | Ref[^25^](#_ENREF_25) |


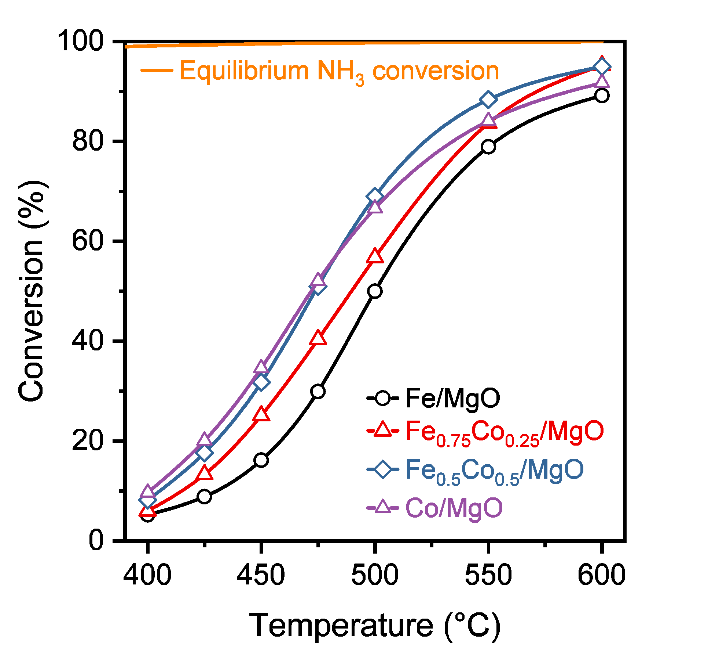


**Supplementary Fig. 17** **Activity screening.** Steady-state NH_3_ conversion of the four Fe_1-x_Co_x_/MgO catalysts during ammonia decomposition. The yellow curve represents the equilibrium conversion of NH_3_ at different temperatures at 1 bar,[^19^](#_ENREF_19) NH_3_ (g) **⇌** 1/2 N_2_ (g)+ 3/2 H_2_ (g), ΔrH^0^_m_ (NH_3_ (g), 298 K) = 46.2 kJ mol-1, Δr G^0^_m_ (NH_3_ (g), 298 K) = −16.63 kJ mol^-1^, ΔCp = 25.46 − 0.01833T + 205000T^−2^.

The NH_3_ conversion values of these four catalysts at 600°C (89-95%) are below the equilibrium NH_3_ conversion (~99%).


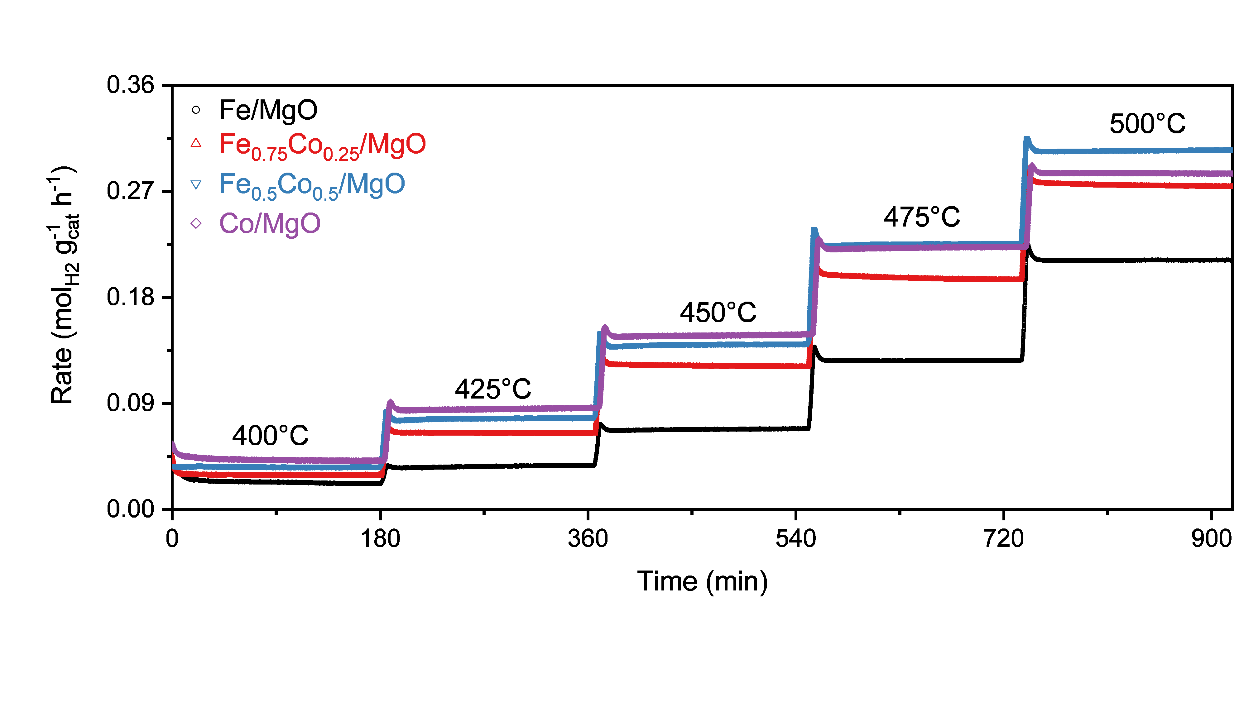


**Supplementary Fig. 18 Kinetic measurements.** Kinetic measurements of ammonia decomposition on four Fe_1-x_Co_x_/MgO catalysts in different temperature regimes under differential conditions.


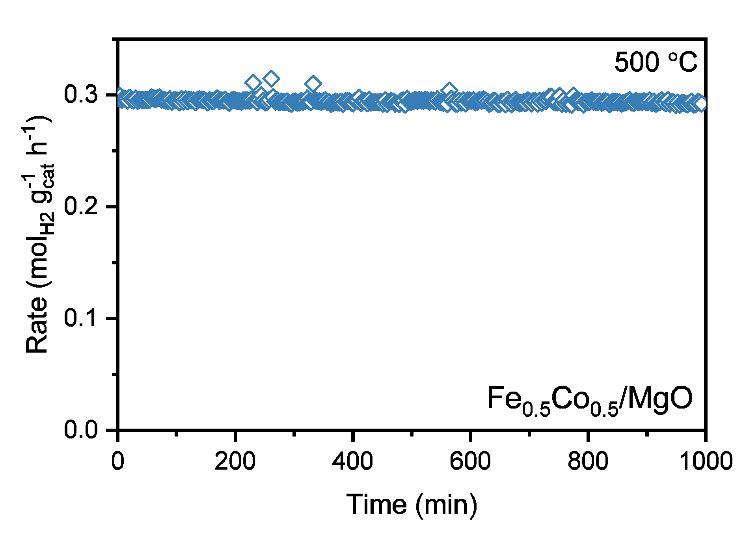


**Supplementary Fig. 19 Stability measurements.** Stability test of Fe_0.5_Co_0.5_/MgO catalyst in ammonia decomposition at 500°C (3%NH_3_, 80 ml min^-1^) for 1000 min.


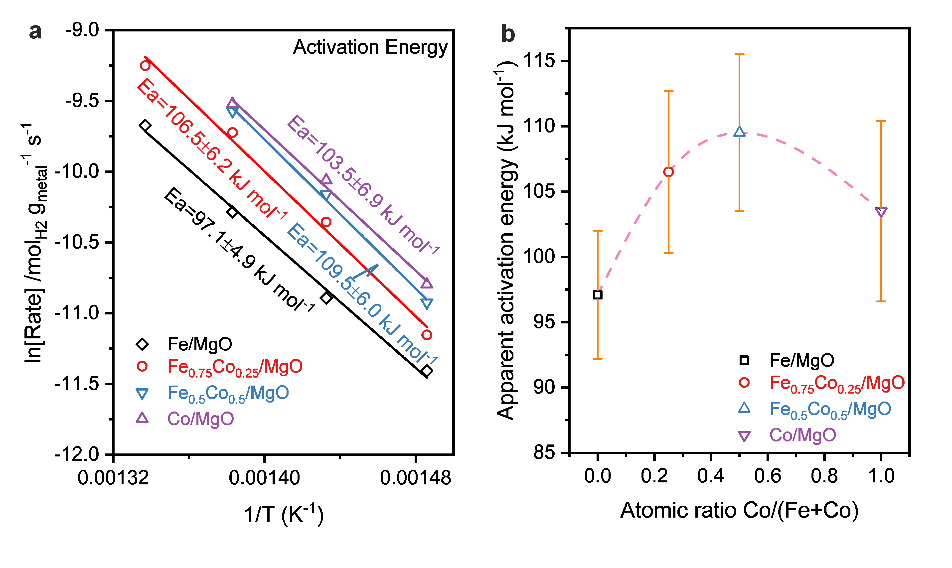


**Supplementary Fig. 20** **Apparent activation energy evaluation.** Arrhenius plots of Fe/MgO, Fe_0.75_Co_0.25_/MgO, Fe_0.5_Co_0.5_/MgO, and Co/MgO catalysts for ammonia decomposition reaction under differential reaction conditions (a). Volcano-like curve for Apparent activation energy of Fe_1-x_Co_x_/MgO catalysts as a function of the atomic ratio of Co/(Fe+Co) (b). The error bars in the figure represents the liner fitting analysis error.


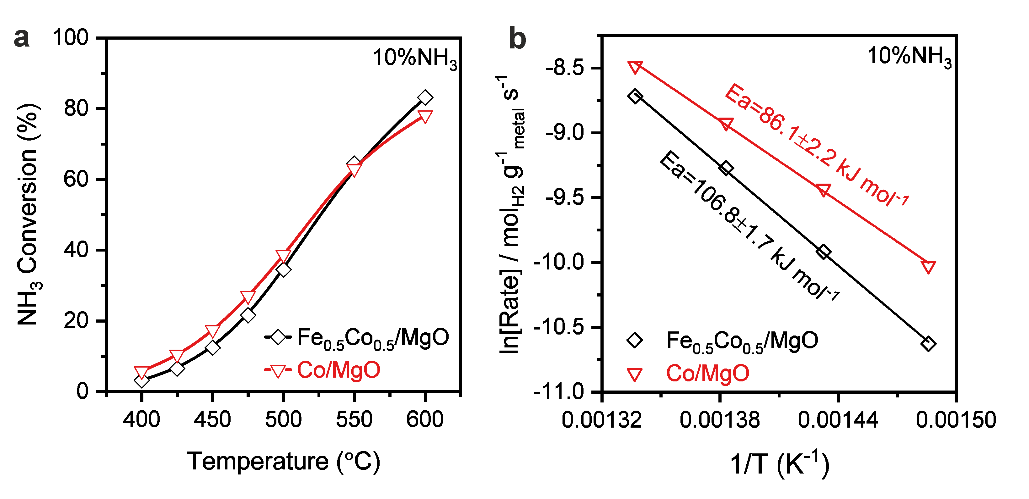


**Supplementary Fig. 21** **Activity difference in 10% NH_3_ reaction gas.** NH_3_ conversion of the Fe_0.5_Co_0.5_/MgO and Co/MgO catalysts in ammonia decomposition in a gas mixture with 10%NH_3_/Ar at a flow rate of 80 ml min^-1^ (a). Arrhenius plots of Fe_0.5_Co_0.5_/MgO, and Co/MgO catalysts for ammonia decomposition in the gas mixture of 10%NH_3_/Ar with flow rate of 80 ml min^-1^ under differential reaction conditions (b). The error bars in the figure represents the liner fitting analysis error.

The NH_3_ conversion for Fe_0.5_Co_0.5_/MgO and Co/MgO was very similar in 3% NH_3_/Ar. (see main text). If measured in 10% NH_3_, the two catalysts are still very similar. Compared to 3%NH_3_, however, the apparent activation energies Co/MgO showed lower E_a_ of 86.1 kJ mol^-1^ while Fe_0.5_Co_0.5_/MgO showed an E_a_ of 106.8 kJ mol^-1^ in the gas mixture of 10% NH_3_, which is similar to the value obtained at 3% NH_3_.


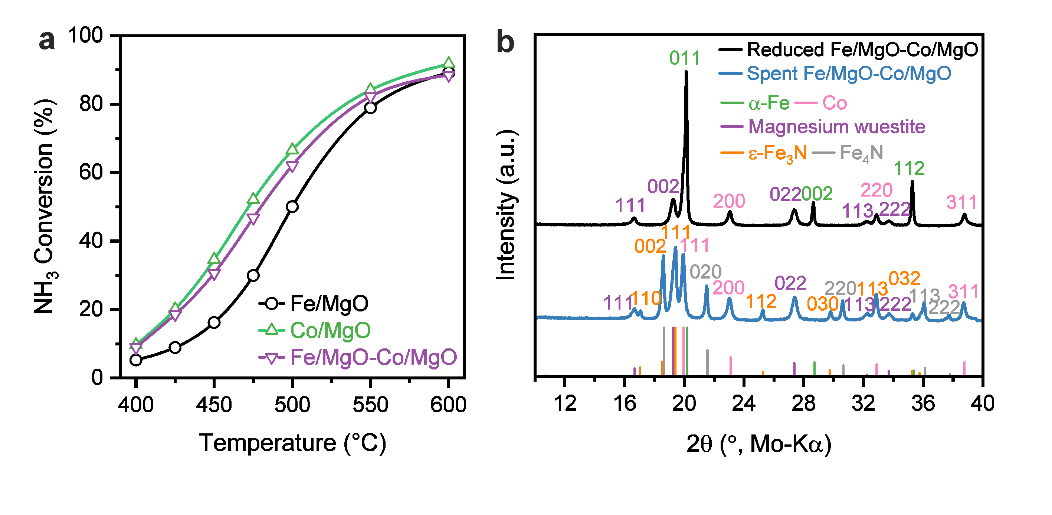


**Supplementary Fig. 22** **Activity test of physically mixed Fe/MgO and Co/MgO.** NH_3_ conversion of physically mixed Fe/MgO (10 mg) – Co/MgO (10 mg) catalyst in ammonia decomposition in a gas mixture of 3%NH_3_/Ar at a flow rate of 80 ml min^-1^, the NH_3_ conversion curves of Fe/MgO and Co/MgO are shown for comparison (a). XRD patterns of reduced physically mixed Fe/MgO – Co/MgO (after isothermal reduction at 600°C) and of the spent mixture (after the ammonia decomposition reaction). (b) References: α-Fe (ICSD: 52258), magnesium wüstite (ICSD: 181215), fcc-cobalt (PDF2: 00-015-0806), Fe_3_N (ICSD: 20389), Fe_4_N (ICSD: 60195)

The activity of physically mixed Fe/MgO (10 mg) – Co/MgO (10 mg) is between the activity of Fe/MgO and Co/MgO catalysts. The XRD pattern of the reduced sample shows only the reflections of magnesium wuestite, α-Fe and fcc Co, indicating no FeCo alloy formation. While the XRD pattern of the spent catalyst shows the reflections of iron nitrides (Fe_3_N and Fe_4_N), magnesium wuestite and fcc Co, indicating clear nitridation of the iron fraction in the mixture. The formation of different nitride species could be related to the presence of active Co/MgO catalyst during the reaction, which changes the local reaction atmosphere towards lower NH_3_/H_2_ ratio.

**8. Metal particle size distribution and H_2_ Chemisorption**


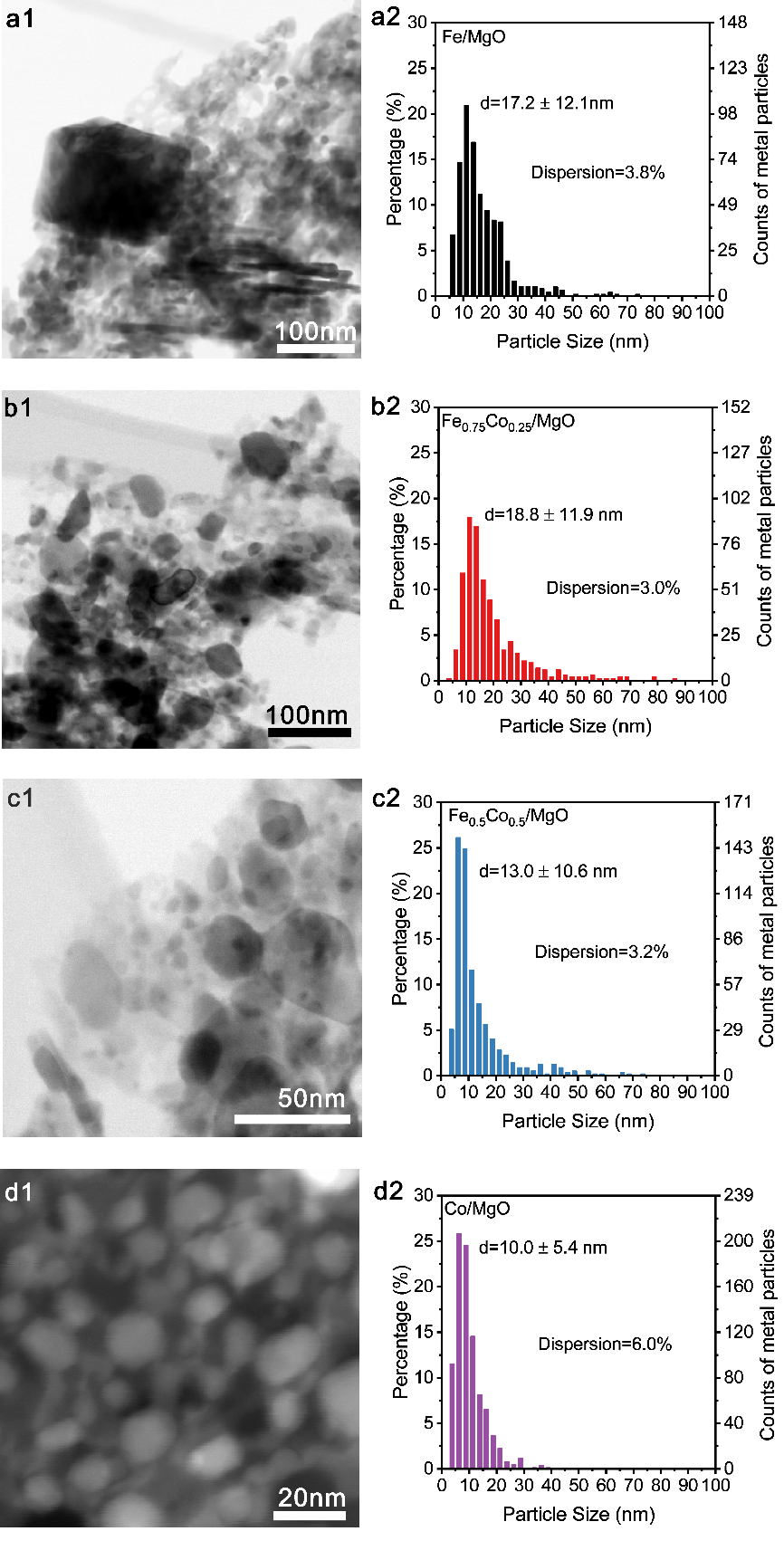


**Supplementary Fig. 23** **Metal particle size distribution.** Representative BF and DF STEM images and the metal particle size distributions of Fe/MgO (a1, a2), Fe_0.75_Co_0.5_/MgO (b1, b2), Fe_0.5_Co_0.5_/MgO (c1, c2), and Co/MgO (d1, d2). The evaluation of metal particle size distribution of these four samples counted more than 400 particles, respectively. The error bar represents the standard deviation through particle size statistical analysis.


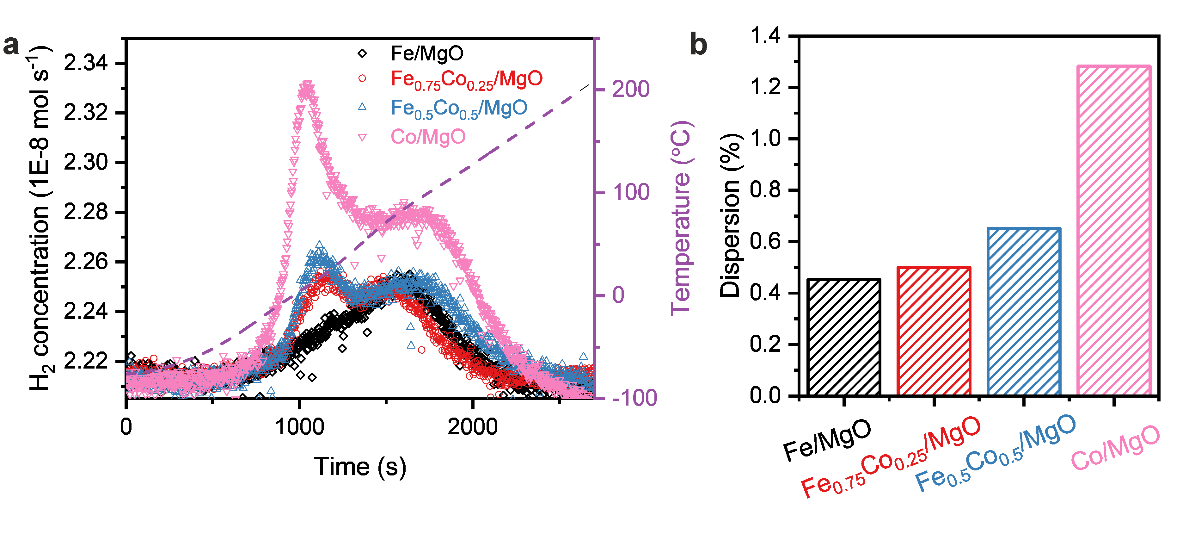


**Supplementary Fig. 24** **H_2_ chemisorption.** H_2_ desorption rate of these four catalysts during TPD after H_2_ chemisorption at -76°C, the purple curve indicated the temperature during TPD (a). The metal dispersion obtained from adsorbed H_2_ molecules during TPD (b).

After H_2_ chemisorption on the catalysts at -76°C for 1h, the TPD in Ar was conducted at a heating rate of 6 K min^-1^. All the Co-containing catalysts showed two clear peaks of H_2_ desorption with the first feature at 10-18°C and the second feature at 83-90°C. The Fe/MgO catalysts only showed one broad peak of H_2_ desorption at 80°C.

**9. Additional DFT information**


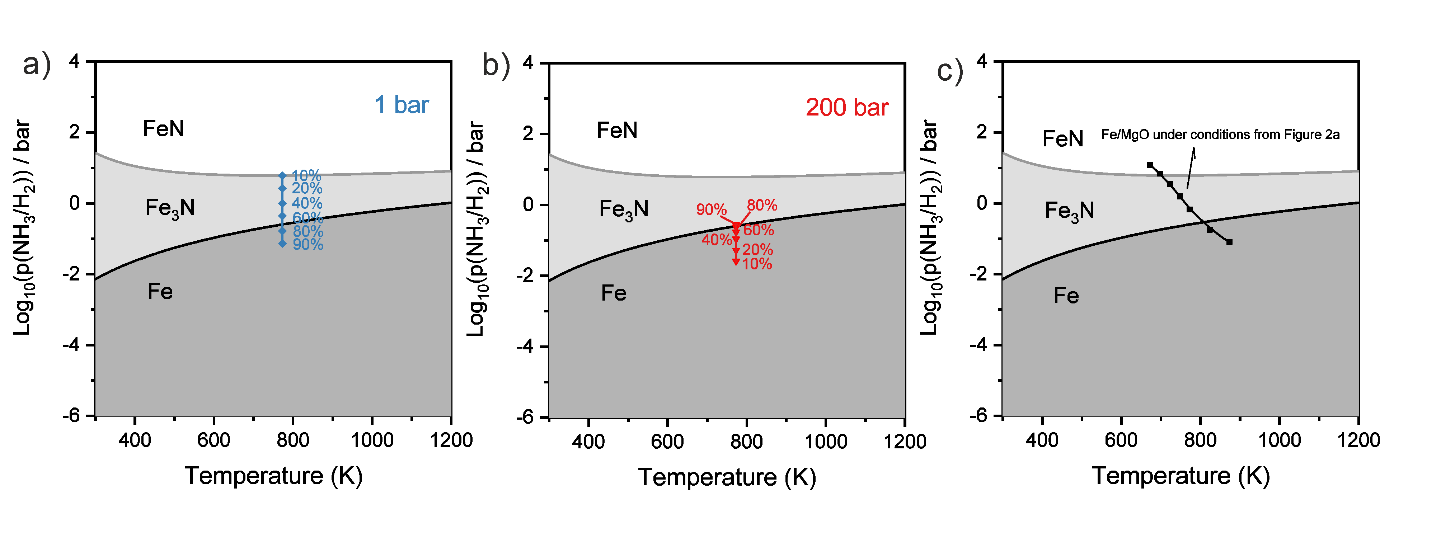


**Supplementary Fig. 25** **Phase diagram.** Calculated phase diagram of nitridation of iron at different temperatures with a) the condition approaching to equilibrium from 10 to 90% for decomposition of NH_3_ at 500 °C under 1 bar, b) the condition approaching to equilibrium from 10 to 90% for reaction of N_2_ and H_2_ (N_2_:H_2_ = 1:3) at 500 °C under 200 bar, and c) Fe/MgO under ammonia decomposition condition at 1 bar in Figure 2a.

**Supplementary Table 6** Optimized lattice constants

|  | Fe | FeN | $\boldsymbol{\epsilon}$ - Fe_3_N | Co | FeCo | Fe_3_Co |
| --- | --- | --- | --- | --- | --- | --- |
| Lattice constants | a=b=c=2.846  $\alpha=\beta=\gamma=90^{\circ}$ | a=b=c=3.002  $\alpha=\beta=\gamma=60^{\circ}$ | a=b= 4.67;  c=4.332  $\alpha=\beta=90^{\circ};$  $\gamma=120^{\circ}$ | a=b= 2.429;  c=4.334  $\alpha=\beta=90^{\circ};$  $\gamma=120^{\circ}$ | a=b=c=2.841  $\alpha=\beta=\gamma=90^{\circ}$ | a=b=c=5.711  $\alpha=\beta=\gamma=90^{\circ}$ |

Structures of the systems used in the Figure 4d: The alloy and nitride structures used in this work are unreconstructed surfaces obtained by truncation of the bulk structure. However, the 210 edge of the stoichiometric FeCo has two types of terminations: a Co rich (66.7% Co) and a Fe rich (66.7% Fe) surface. The N binding energies on these two terminations differ by about 0.4 eV in favor of the Fe rich terminations. Additional Co and Fe segregation to the surface were tested with and without N adsorption. We found that both, the segregation of Co and Fe are unfavorable by +0.2 eV and +0.45 eV, respectively. We therefore chose Fe rich terminations for the stoichiometric FeCo(210) and Fe_3_Co(210) as adsorption sites.


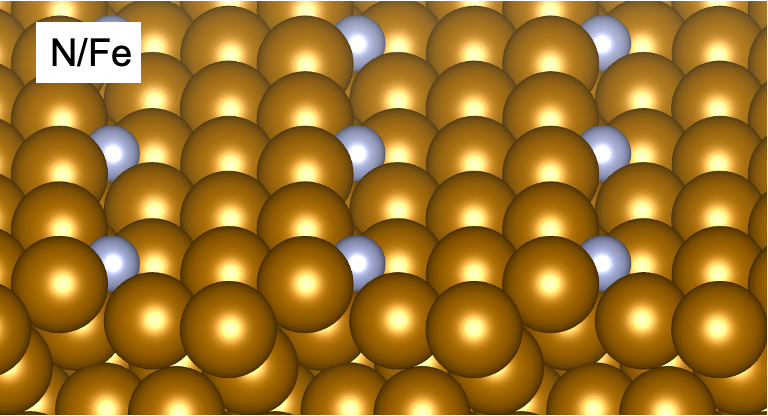


Fe N

1.0000000000000000

6.3638494640000003 0.0000000000000000 0.0000000000000000

0.0000000000000000 5.6920000000000002 0.0000000000000000

0.0000000000000000 0.0000000000000000 22.9777782700000017

24 1

Selective dynamics

Cartesian

0.0000000000000000 0.0000000000000000 7.9887719298211319 F F F

0.0000000000000000 2.8460000000000001 7.9887719298211319 F F F

1.9091548391999824 1.4230000000000000 8.6251568762171864 F F F

1.9091548391999824 4.2690000000000001 8.6251568762171864 F F F

5.7274645175999916 4.2690000000000001 9.8979267690096222 F F F

5.7274645175999916 1.4230000000000000 9.8979267690096222 F F F

3.8183096784000092 0.0000000000000000 9.2615418226134025 F F F

3.8183096784000092 2.8460000000000001 9.2615418226134025 F F F

1.2727698928000177 2.8460000000000001 10.5343117154056767 F F F

1.2727698928000177 0.0000000000000000 10.5343117154056767 F F F

3.1819247320000001 1.4230000000000000 11.1706966618018928 F F F

3.1819247320000001 4.2690000000000001 11.1706966618018928 F F F

5.0702880412201354 5.6919551479734496 11.8832456188083828 T T T

5.0814640218915912 2.8459868498081997 11.8498583031296061 T T T

0.6143765895568227 1.4146062956641456 12.4977291865430242 T T T

0.6144158842617388 4.2773463003121837 12.4977363198953793 T T T

4.4450949750545599 4.2419536154699085 13.8522214869997331 T T T

2.5107521936597901 2.8459907857024955 13.0079319363170605 T T T

4.4451585307752941 1.4499238390847802 13.8521927694998865 T T T

2.5563008194868595 0.0000025789845284 13.0975988289492200 T T T

0.0558000310333649 5.6919304981359549 14.4326537054513295 T T T

2.0441202423674403 1.4382822399228870 14.9511449719136351 T T T

0.0666597743235747 2.8459695015752198 14.4300626617714691 T T T

2.0440663635147791 4.2535778830098456 14.9511011292173350 T T T

3.3925215791071679 2.8459297534674515 14.7584820627076194 T T T


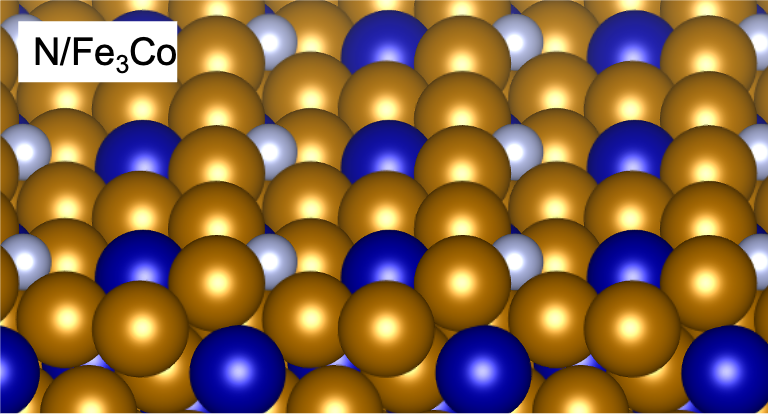


Co Fe N

1.0000000000000000

6.3850921100000004 0.0000000000000000 0.0000000000000000

0.0000000000000000 5.7110000000000003 0.0000000000000000

0.0000000000000000 0.0000000000000000 23.0544785099999991

6 18 1

Selective dynamics

Cartesian

0.0000000000000000 0.0000000000000000 8.0154385952020277 F F F

3.8310552660000092 2.8555000000000001 9.2924570169466989 F F F

1.2770184220000178 2.8555000000000001 10.5694754386913736 F F F

5.1063730907040412 2.8555186174309055 11.8808173462530551 T T T

2.5658380638145566 0.0001497333667143 13.1337156835353888 T T T

0.1098954451079247 2.8555862591098453 14.3407332092614581 T T T

0.0000000000000000 2.8555000000000001 8.0154385952020277 F F F

1.9155276329999824 1.4277500000000001 8.6539478060742816 F F F

1.9155276329999824 4.2832500000000007 8.6539478060742816 F F F

5.7465828989999910 4.2832500000000007 9.9309662278191198 F F F

5.7465828989999910 1.4277500000000001 9.9309662278191198 F F F

3.8310552660000092 0.0000000000000000 9.2924570169466989 F F F

1.2770184220000178 0.0000000000000000 10.5694754386913736 F F F

3.1925460550000002 1.4277500000000001 11.2079846495637909 F F F

3.1925460550000002 4.2832500000000007 11.2079846495637909 F F F

5.0858126528865677 0.0001011041888001 11.9172915527353744 T T T

0.0417682975768538 0.0000205335816657 14.4564036900354438 T T T

2.5226771595515936 2.8555196221764914 13.0368406071136160 T T T

0.6206940948509038 1.4124787785716428 12.5029594704539235 T T T

4.4616321000732668 1.4727522057676983 13.8846475472650557 T T T

2.0186144523566827 1.4495522700710390 14.9827291272154248 T T T

0.6208012517168642 4.2986902751553577 12.5029299293414145 T T T

4.4615459637288986 4.2385220466182227 13.8845132692268205 T T T

2.0188686919321519 4.2616925976620461 14.9826607613821903 T T T

3.3806819459422099 2.8555461027406293 14.8206743974155941 T T T


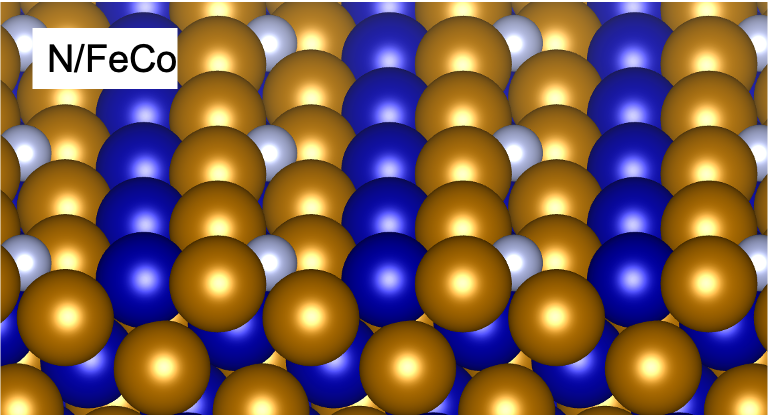


Co Fe N

1.0000000000000000

6.3526691240000002 0.0000000000000000 0.0000000000000000

0.0000000000000000 5.6820000000000004 0.0000000000000000

0.0000000000000000 0.0000000000000000 22.9374097200000016

12 12 1

Selective dynamics

Cartesian

0.0000000000000000 0.0000000000000000 7.9747368418636233 F F F

0.0000000000000000 2.8410000000000002 7.9747368418636233 F F F

3.8116014744000091 2.8410000000000002 9.2452706666404421 F F F

3.8116014744000091 0.0000000000000000 9.2452706666404421 F F F

1.2705338248000178 0.0000000000000000 10.5158044914172653 F F F

1.2705338248000178 2.8410000000000002 10.5158044914172653 F F F

5.0668635817299164 5.6797352864475217 11.8533770985895810 T T T

5.0832157621968719 2.8393289019080852 11.8351416673269565 T T T

2.5308683364220803 2.8390582774378572 12.9643708489884766 T T T

2.5853531411313604 5.6782176426279740 13.0697706310257598 T T T

0.0932358799204799 5.6787638798823830 14.2643768546253860 T T T

0.1719128716678319 2.8372385726233009 14.2626951364206462 T T T

1.9058007371999823 1.4205000000000001 8.6100037542519523 F F F

1.9058007371999823 4.2614999999999998 8.6100037542519523 F F F

5.7174022115999907 4.2614999999999998 9.8805375790289354 F F F

5.7174022115999907 1.4205000000000001 9.8805375790289354 F F F

3.1763345620000001 1.4205000000000001 11.1510714038057550 F F F

3.1763345620000001 4.2614999999999998 11.1510714038057550 F F F

0.6269409815142049 1.4003645607476944 12.4357339641561868 T T T

0.6265813476898850 4.2768860056657632 12.4358706436144129 T T T

4.4677186262969020 4.2238858337824299 13.8490825012959977 T T T

4.4677473138440575 1.4514925053118199 13.8482828822025397 T T T

2.0166008298837950 1.4244944377523976 14.9302021582125466 T T T

2.0167806995929491 4.2500738595135807 14.9314825088748702 T T T

3.3746129969532830 2.8371143912782499 14.7743334839884355 T T T


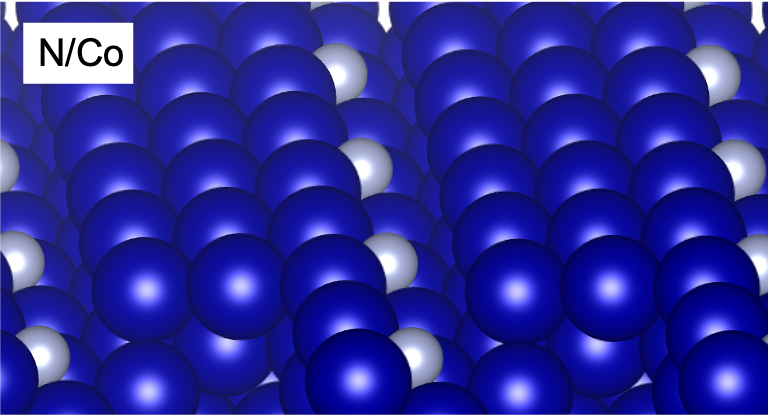


Co N

1.0000000000000000

9.7090132390860457 0.0000000000000000 0.0000000000000000

-2.4272533097715114 4.2041260553639743 0.0000000000000000

0.0000000000000000 0.0000000000000000 22.4964720938002216

30 1

Selective dynamics

Cartesian

0.0000000000000000 0.0000000000000000 7.9999999999999760 F F F

-1.2136266548857557 2.1020630276819872 7.9999999999999760 F F F

2.4272533097715114 0.0000000000000000 7.9999999999999760 F F F

1.2136266548857557 2.1020630276819872 7.9999999999999760 F F F

4.8545066195430229 0.0000000000000000 7.9999999999999760 F F F

3.6408799646572669 2.1020630276819872 7.9999999999999760 F F F

7.2817599293145339 0.0000000000000000 7.9999999999999760 F F F

6.0681332744287779 2.1020630276819872 7.9999999999999760 F F F

6.0681332744288135 3.5034383794699586 10.1654906979334516 F F F

7.2817599293145694 1.4013753517879715 10.1654906979334516 F F F

3.6408799646573016 3.5034383794699586 10.1654906979334516 F F F

4.8545066195430575 1.4013753517879715 10.1654906979334516 F F F

1.2136266548857901 3.5034383794699586 10.1654906979334516 F F F

2.4272533097715456 1.4013753517879715 10.1654906979334516 F F F

-1.2136266548857213 3.5034383794699586 10.1654906979334516 F F F

0.0000000000000344 1.4013753517879715 10.1654906979334516 F F F

7.2724326015724774 4.1979741332640756 12.2363714748509054 T T T

8.4833406367555018 2.1000951173527360 12.2353228868091044 T T T

2.4245815759950822 0.0016892506650789 12.2443729150285581 T T T

1.1914622815782461 2.0913866972542423 12.2124025139076586 T T T

4.8873880418240985 0.0275329282418237 12.3172229599736323 T T T

3.6794905766735608 2.1199372387687183 12.3196422370874590 T T T

7.2831246822527635 0.0021493832629996 12.1531050034084878 T T T

6.0552630418029603 2.0964334970349960 12.1613189625846445 T T T

-1.2865977933202659 3.4633100899316522 14.2198849431586485 T T T

-0.0493726944704328 1.3764394347092954 14.2336014420247352 T T T

1.1563301374109121 3.5036610601042795 14.2407894948241633 T T T

2.3922941702809437 1.3548602170695472 14.2430761654966727 T T T

5.9667485344414430 3.4482048087304982 14.1432906505539950 T T T

7.1816165804375078 1.3410840022314225 14.1417632565524070 T T T

2.9352045305167644 3.0951348253149180 13.8133376099736065 T T T


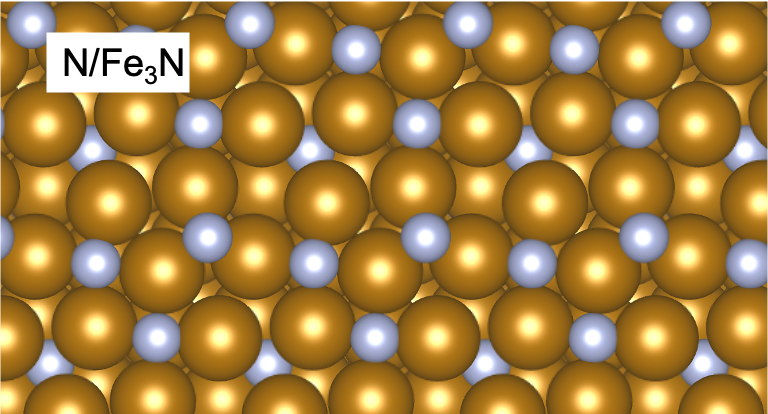


Fe N

1.0000000000000000

6.3695745692570940 0.0000000000000000 0.0000000000000000

1.2346633322030995 6.2487668102946383 0.0000000000000000

0.0000000000000000 0.0000000000000000 23.3971941178139460

24 9

Selective dynamics

Cartesian

6.6873357793319723 3.7920255981425943 8.0490199439712224 F F F

5.8120230679204292 1.2443504991567551 8.3581893055148040 F F F

4.1003562062994909 3.3695947431551181 8.4076222252664596 F F F

5.0169397121638291 5.8258025530321564 8.0486443210016763 F F F

2.0297479010176445 1.6679873207355675 8.0000000000000391 F F F

2.3475518878950012 5.4606698876412549 8.3581593991942871 F F F

3.7410353681108552 1.3709093125433942 10.1043033772597237 F F F

4.1003859889024117 5.0720010238521942 10.4134727388033017 F F F

1.1540557950783745 0.9484784575559184 10.4629056585551279 F F F

2.0706393009427666 3.4046862674330014 10.1039277542901758 F F F

6.6876853912567222 5.4956378454310064 10.0552834332885386 F F F

5.7708260459309786 3.0395536020420555 10.4134428324827866 F F F

1.9984242698241486 5.2222241409164614 12.2110377711302309 T T T

1.1192539356501690 2.6427736649730393 12.5156878209887257 T T T

5.7947648652269343 4.7624307691280778 12.4944176862384904 T T T

5.5251418587228009 1.0298458144277516 12.1468705299573561 T T T

3.7693187657755809 3.0870284342343925 12.0991743049929070 T T T

2.8505087709992325 0.6321214887061294 12.4698447850693768 T T T

5.5262053854448947 2.7631803729135274 14.2135748915341011 T T T

4.7065711672749071 0.1465261652827874 14.3597654906569066 T T T

2.7988281315320762 2.4404203130307747 14.3552112277510222 T T T

3.7881360159329818 4.8254062099853119 14.2083612260530714 T T T

0.8609255966070187 0.6010571217220319 14.2681004768919735 T T T

1.1239229747568891 4.4072528089189325 14.5679140190147116 T T T

2.2091924028978758 3.5603837592095329 8.2036659229342241 F F F

2.4478212861440531 0.2665555535155973 9.2313438179482414 F F F

5.6324665609338531 1.1392674736103334 10.2589493562228888 F F F

7.1057587763831851 4.0942060782110810 11.2866272512367392 F F F

3.8778134536452593 4.9963612682110430 12.3219244254923659 T T T

4.2019355922988382 1.6800171000164859 13.4237444712072858 T T T

0.9822473713044245 2.4497218372805318 14.6100836085055672 T T T

5.5603919466052432 4.6246492521534774 14.7427234265111267 T T T

2.4566631482292025 5.3772188564773975 15.2783445307602328 T T T

Fe

1.0000000000000000

6.3638494640000003 0.0000000000000000 0.0000000000000000

0.0000000000000000 5.6920000000000002 0.0000000000000000

0.0000000000000000 0.0000000000000000 22.9777782700000017

24

Selective dynamics

Cartesian

0.0000000000000000 0.0000000000000000 7.9887719298211319 F F F

0.0000000000000000 2.8460000000000001 7.9887719298211319 F F F

1.9091548391999824 1.4230000000000000 8.6251568762171864 F F F

1.9091548391999824 4.2690000000000001 8.6251568762171864 F F F

3.8183096784000092 2.8460000000000001 9.2615418226134025 F F F

3.8183096784000092 0.0000000000000000 9.2615418226134025 F F F

5.7274645175999916 1.4230000000000000 9.8979267690096222 F F F

5.7274645175999916 4.2690000000000001 9.8979267690096222 F F F

1.2727698928000177 0.0000000000000000 10.5343117154056767 F F F

1.2727698928000177 2.8460000000000001 10.5343117154056767 F F F

3.1819247320000001 1.4230000000000000 11.1706966618018928 F F F

3.1819247320000001 4.2690000000000001 11.1706966618018928 F F F

5.0789331959729038 5.6919676664498411 11.8612097643539638 T T T

5.0787734194395471 2.8459768155705727 11.8617446944600964 T T T

0.6212622961789404 1.4230735263427079 12.4949180842732819 T T T

0.6211621590930213 4.2689145428633823 12.4948910967548379 T T T

2.5579946358259429 5.6919780255767716 13.0964468564861818 T T T

2.5579301783737969 2.8459485776642426 13.0972531344241023 T T T

4.4635665519780297 1.4226706618782914 13.8306416192158448 T T T

4.4634580643637074 4.2693274342198961 13.8306834396000919 T T T

0.0571867121096115 0.0000030344932854 14.4271274453633467 T T T

0.0567166084653165 2.8460094684255077 14.4268402858793419 T T T

2.0204725896243008 4.2691176243939744 14.9502983618170671 T T T

2.0205004339813315 1.4227821271222234 14.9503023724789426 T T T

Fe N

1.0000000000000000

6.3695745692570940 0.0000000000000000 0.0000000000000000

1.2346633322030995 6.2487668102946383 0.0000000000000000

0.0000000000000000 0.0000000000000000 23.3971941178139460

24 8

Selective dynamics

Cartesian

6.6873357793319714 3.7920255981425943 8.0490199439712224 F F F

5.8120230679204292 1.2443504991567551 8.3581893055148040 F F F

4.1003562062994909 3.3695947431551181 8.4076222252664596 F F F

5.0169397121638291 5.8258025530321564 8.0486443210016763 F F F

2.0297479010176445 1.6679873207355675 8.0000000000000391 F F F

2.3475518878950012 5.4606698876412549 8.3581593991942871 F F F

3.7410353681108552 1.3709093125433942 10.1043033772597237 F F F

4.1003859889024117 5.0720010238521942 10.4134727388033017 F F F

1.1540557950783745 0.9484784575559184 10.4629056585551279 F F F

2.0706393009427666 3.4046862674330014 10.1039277542901758 F F F

6.6876853912567222 5.4956378454310064 10.0552834332885386 F F F

5.7708260459309786 3.0395536020420555 10.4134428324827866 F F F

1.9901865501137714 5.2144306810736065 12.2033860528502718 T T T

1.1235237488444472 2.6572877325204756 12.5116050478688763 T T T

5.7862254571388050 4.7619140743010133 12.4861083501868766 T T T

5.5198296137698941 1.0258330869465655 12.1476978115932859 T T T

3.7444098449421905 3.0654615770524272 12.0772398355221871 T T T

2.8408268623133477 0.6087975344078599 12.5179725488571201 T T T

5.5286911300349049 2.7782239111195541 14.2427470466950705 T T T

4.6975518343366858 0.1581016930998154 14.3753398160837751 T T T

2.8291125556132894 2.4719587386881980 14.3816269377873542 T T T

3.7571857947701233 4.7645234253537181 14.1894980341261512 T T T

0.7964695578394996 0.6028424869131824 14.1143186525081425 T T T

1.1210665962444584 4.4094912705776412 14.4797309191998806 T T T

2.2091924028978753 3.5603837592095329 8.2036659229342241 F F F

2.4478212861440531 0.2665555535155973 9.2313438179482414 F F F

5.6324665609338531 1.1392674736103334 10.2589493562228888 F F F

7.1057587763831851 4.0942060782110810 11.2866272512367392 F F F

3.8669615657150418 4.9800831409698834 12.2938414144917925 T T T

4.2032877519984195 1.6808697317944887 13.4311775546078369 T T T

1.0079868707231099 2.4460015990357480 14.6022967932889092 T T T

5.5693477601677124 4.6516414151496859 14.7634958688111713 T T T

Fe N

1.0000000000000000

5.7639810245520664 0.0000000000000000 0.0000000000000000

0.0000000000000006 8.6631741532934612 0.0000000000000000

0.0000000000000000 0.0000000000000000 29.7167061994824167

24 24

Selective dynamics

Cartesian

0.0000000000000003 0.0000000000000000 10.8877276209436662 F F F

2.8819905122760332 0.0000000000000000 10.8877276209436662 F F F

0.0000000000000005 2.8877247177644874 10.8877276209436662 F F F

2.8819905122760332 2.8877247177644874 10.8877276209436662 F F F

0.0000000000000007 5.7754494355289747 10.8877276209436662 F F F

2.8819905122760336 5.7754494355289747 10.8877276209436662 F F F

0.0000000000000009 5.7754494355289747 13.7754552418873324 F F F

2.8819905122760336 5.7754494355289747 13.7754552418873324 F F F

0.0000000000000007 2.8877247177644874 13.7754552418873324 F F F

2.8819905122760332 2.8877247177644874 13.7754552418873324 F F F

0.0000000000000005 0.0000000000000000 13.7754552418873324 F F F

2.8819905122760332 0.0000000000000000 13.7754552418873324 F F F

0.0000000000000008 0.0000000000000000 16.6631828628310004 T T T

2.8819905122760336 0.0000000000000000 16.6631828628310004 T T T

0.0000000000000010 2.8877247177644874 16.6631828628310004 T T T

2.8819905122760336 2.8877247177644874 16.6631828628310004 T T T

0.0000000000000012 5.7754494355289747 16.6631828628310004 T T T

2.8819905122760341 5.7754494355289747 16.6631828628310004 T T T

0.0000000000000010 0.0000000000000000 19.5509104837746648 T T T

2.8819905122760336 0.0000000000000000 19.5509104837746648 T T T

0.0000000000000012 2.8877247177644874 19.5509104837746648 T T T

2.8819905122760336 2.8877247177644874 19.5509104837746648 T T T

0.0000000000000014 5.7754494355289747 19.5509104837746648 T T T

2.8819905122760341 5.7754494355289747 19.5509104837746648 T T T

4.3229857684140498 2.1657935383233649 10.1657957157077501 F F F

4.3229857684140498 5.0535182560878518 10.1657957157077501 F F F

4.3229857684140498 7.9412429738523400 10.1657957157077501 F F F

1.4409952561380168 0.7219311794411218 11.6096595261795841 F F F

1.4409952561380170 3.6096558972056094 11.6096595261795841 F F F

1.4409952561380173 6.4973806149700968 11.6096595261795841 F F F

4.3229857684140507 7.9412429738523400 13.0535233366514163 F F F

4.3229857684140507 5.0535182560878518 13.0535233366514163 F F F

4.3229857684140507 2.1657935383233649 13.0535233366514163 F F F

1.4409952561380177 6.4973806149700968 14.4973871471232503 F F F

1.4409952561380175 3.6096558972056094 14.4973871471232503 F F F

1.4409952561380173 0.7219311794411218 14.4973871471232503 F F F

4.3229857684140507 2.1657935383233649 15.9412509575950843 T T T

4.3229857684140507 5.0535182560878518 15.9412509575950843 T T T

4.3229857684140507 7.9412429738523400 15.9412509575950843 T T T

1.4409952561380173 0.7219311794411218 17.3851147680669165 T T T

1.4409952561380175 3.6096558972056094 17.3851147680669165 T T T

1.4409952561380177 6.4973806149700968 17.3851147680669165 T T T

4.3229857684140507 2.1657935383233649 18.8289785785387522 T T T

4.3229857684140507 5.0535182560878518 18.8289785785387522 T T T

4.3229857684140507 7.9412429738523400 18.8289785785387522 T T T

1.4409952561380175 0.7219311794411218 20.2728423890105809 T T T

1.4409952561380177 3.6096558972056094 20.2728423890105809 T T T

1.4409952561380179 6.4973806149700968 20.2728423890105809 T T T

**10. Supplementary References**

1. Fogler, H.S. *Elements of chemical reaction engineering*, (Prentice Hall Professional Technical Reference, 2006).

2. Mears, D.E. Tests for transport limitations in experimental catalytic reactors. *Ind. Eng. Chem. Proc. Dd.* **10**, 541-547 (1971).

3. Usman, M. *et al.* Magnetite and green rust: Synthesis, properties, and environmental applications of mixed-valent iron minerals. *Chem. Rev.* **118**, 3251-3304 (2018).

4. Lee, N., Petrenko, T., Bergmann, U., Neese, F. & DeBeer, S. Probing valence orbital composition with iron kβ X-ray emission spectroscopy. *J. Am. Chem. Soc.* **132**, 9715-9727 (2010).

5. Pollock, C.J. & DeBeer, S. Insights into the geometric and electronic structure of transition metal centers from valence-to-core X-ray emission spectroscopy. *Acc. Chem. Res.* **48**, 2967-2975 (2015).

6. Lancaster, K.M. *et al.* X-ray emission spectroscopy evidences a central carbon in the nitrogenase iron-molybdenum cofactor. *Science* **334**, 974-977 (2011).

7. Pollock, C.J. & DeBeer, S. Valence-to-core X-ray emission spectroscopy: A sensitive probe of the nature of a bound ligand. *J. Am. Chem. Soc.* **133**, 5594-5601 (2011).

8. Bergmann, U., Horne, C.R., Collins, T.J., Workman, J.M. & Cramer, S.P. Chemical dependence of interatomic X-ray transition energies and intensities – a study of mn kβ″ and kβ_2,5_ spectra. *Chem. Phys. Lett.* **302**, 119-124 (1999).

9. Ravel, B. & Newville, M. Athena, artemis, hephaestus: Data analysis for X-ray absorption spectroscopy using ifeffit. *J. Synchrotron Radiat.* **12**, 537-541 (2005).

10. Grunwaldt, J.-D., Vegten, N.v. & Baiker, A. Insight into the structure of supported palladium catalysts during the total oxidation of methane. *Chem. Commun.*, 4635-4637 (2007).

11. Wyckoff, R.W.G. Interscience publishers, new york, new york rocksalt structure. *Crystal structures* **1**, 7-83 (1963).

12. Passerini, L. Ricerche sugli spinelli. Ii. I composti: CuAl_2_O_4_; MgAl_2_O_4_; MgFe_2_O_4_; ZnAl_2_O_4_; ZnCr_2_O_4_; ZnFe_2_O_4_; MnFe_2_O_4_. *Gazz Chim Ital* **60**, 389-399 (1930).

13. Wyckoff, R.W.G. Interscience publishers, new york, new york rocksalt structure. *Crystal structures* **1**, 85-237 (1963).

14. Suzuki, K., Morita, H., Kaneko, T., Yoshida, H. & Fujimori, H. Crystal structure and magnetic properties of the compound fen. *J. Alloys Compd.* **201**, 11-16 (1993).

15. Jack, K. The iron-nitrogen system: The crystal structures of [epsilon]-phase iron nitrides. *Acta Crystallogr.* **5**, 404-411 (1952).

16. Bayliss, P. Revised unit-cell dimensions, space group, and chemical formula of some metallic minerals. *Can. Mineral.* **28**, 751-755 (1990).

17. Grazulis, S. *et al.* Crystallography open database - an open-access collection of crystal structures. *J. Appl. Crystallogr.* **42**, 726-729 (2009).

18. Gražulis, S., Merkys, A. & Vaitkus, A. Crystallography open database (cod). in *Handbook of materials modeling : Methods: Theory and modeling* (eds. Andreoni, W. & Yip, S.) 1-19 (Springer International Publishing, Cham, 2018).

19. Yin, S.-F. *et al.* Investigation on the catalysis of CO_x_-free hydrogen generation from ammonia. *J. Catal.* **224**, 384-396 (2004).

20. Gu, Y.-Q. *et al.* Transition metal nanoparticles dispersed in an alumina matrix as active and stable catalysts for CO_x_-free hydrogen production from ammonia. *J. Mater. Chem. A* **3**, 17172-17180 (2015).

21. Duan, X. *et al.* Tuning the size and shape of Fe nanoparticles on carbon nanofibers for catalytic ammonia decomposition. *Appl. Catal. B: Environ.* **101**, 189-196 (2011).

22. Lu, A.-H. *et al.* Spatially and size selective synthesis of Fe-based nanoparticles on ordered mesoporous supports as highly active and stable catalysts for ammonia decomposition. *J. Am. Chem. Soc.* **132**, 14152-14162 (2010).

23. Lorenzut, B., Montini, T., Bevilacqua, M. & Fornasiero, P. FeMo-based catalysts for H_2_ production by NH_3_ decomposition. *Appl. Catal. B: Environ.* **125**, 409-417 (2012).

24. Ju, X. *et al.* Mesoporous Ru/MgO prepared by a deposition-precipitation method as highly active catalyst for producing CO_x_-free hydrogen from ammonia decomposition. *Appl. Catal. B: Environ.* **211**, 167-175 (2017).

25. Yin, S.F., Xu, B.Q., Wang, S.J., Ng, C.F. & Au, C.T. Magnesia–carbon nanotubes (MgO–CNTs) nanocomposite: Novel support of Ru catalyst for the generation of CO_x_-free hydrogen from ammonia. *Catal. Lett.* **96**, 113-116 (2004).
